# High-speed flexible near-infrared organic photodiode for optical communication

**DOI:** 10.1093/nsr/nwad311

**Published:** 2023-12-11

**Authors:** Yu Zhu, Hongbin Chen, Ruiman Han, Hao Qin, Zhaoyang Yao, Hang Liu, Yanfeng Ma, Xiangjian Wan, Guanghui Li, Yongsheng Chen

**Affiliations:** The Centre of Nanoscale Science and Technology and Key Laboratory of Functional Polymer Materials, Institute of Polymer Chemistry, Tianjin Key Laboratory of Functional Polymer Materials, College of Chemistry, and Renewable Energy Conversion and Storage Center (RECAST), Nankai University, Tianjin 300071, China; The Centre of Nanoscale Science and Technology and Key Laboratory of Functional Polymer Materials, Institute of Polymer Chemistry, Tianjin Key Laboratory of Functional Polymer Materials, College of Chemistry, and Renewable Energy Conversion and Storage Center (RECAST), Nankai University, Tianjin 300071, China; The Centre of Nanoscale Science and Technology and Key Laboratory of Functional Polymer Materials, Institute of Polymer Chemistry, Tianjin Key Laboratory of Functional Polymer Materials, College of Chemistry, and Renewable Energy Conversion and Storage Center (RECAST), Nankai University, Tianjin 300071, China; The Centre of Nanoscale Science and Technology and Key Laboratory of Functional Polymer Materials, Institute of Polymer Chemistry, Tianjin Key Laboratory of Functional Polymer Materials, College of Chemistry, and Renewable Energy Conversion and Storage Center (RECAST), Nankai University, Tianjin 300071, China; The Centre of Nanoscale Science and Technology and Key Laboratory of Functional Polymer Materials, Institute of Polymer Chemistry, Tianjin Key Laboratory of Functional Polymer Materials, College of Chemistry, and Renewable Energy Conversion and Storage Center (RECAST), Nankai University, Tianjin 300071, China; The Centre of Nanoscale Science and Technology and Key Laboratory of Functional Polymer Materials, Institute of Polymer Chemistry, Tianjin Key Laboratory of Functional Polymer Materials, College of Chemistry, and Renewable Energy Conversion and Storage Center (RECAST), Nankai University, Tianjin 300071, China; The Centre of Nanoscale Science and Technology and Key Laboratory of Functional Polymer Materials, Institute of Polymer Chemistry, Tianjin Key Laboratory of Functional Polymer Materials, College of Chemistry, and Renewable Energy Conversion and Storage Center (RECAST), Nankai University, Tianjin 300071, China; The Centre of Nanoscale Science and Technology and Key Laboratory of Functional Polymer Materials, Institute of Polymer Chemistry, Tianjin Key Laboratory of Functional Polymer Materials, College of Chemistry, and Renewable Energy Conversion and Storage Center (RECAST), Nankai University, Tianjin 300071, China; The Centre of Nanoscale Science and Technology and Key Laboratory of Functional Polymer Materials, Institute of Polymer Chemistry, Tianjin Key Laboratory of Functional Polymer Materials, College of Chemistry, and Renewable Energy Conversion and Storage Center (RECAST), Nankai University, Tianjin 300071, China; The Centre of Nanoscale Science and Technology and Key Laboratory of Functional Polymer Materials, Institute of Polymer Chemistry, Tianjin Key Laboratory of Functional Polymer Materials, College of Chemistry, and Renewable Energy Conversion and Storage Center (RECAST), Nankai University, Tianjin 300071, China; State Key Laboratory of Elemento-Organic Chemistry, Nankai University, Tianjin 300071, China

**Keywords:** flexible organic photodetector, high-speed NIR organic photodetector, optical communication, light fidelity, narrow-bandgap non-fullerene acceptors

## Abstract

Optical communication is a particularly compelling technology for tackling the speed and capacity bottlenecks in data communication in modern society. Currently, the silicon photodetector plays a dominant role in high-speed optical communication across the visible-near-infrared spectrum. However, its intrinsic rigid structure, high working bias and low responsivity essentially limit its application in next-generation flexible optoelectronic devices. Herein, we report a narrow-bandgap non-fullerene acceptor (NFA) with a remarkable π-extension in the direction of both central and end units (CH17) with respect to the Y6 series, which demonstrates a more effective and compact 3D molecular packing, leading to lower trap states and energetic disorders in the photoactive film. Consequently, the optimized solution-processed organic photodetector (OPD) with CH17 exhibits a remarkable response time of 91 ns (λ = 880 nm) due to the high charge mobility and low parasitic capacitance, exceeding the values of most commercial Si photodiodes and all NFA-based OPDs operating in self-powered mode. More significantly, the flexible OPD exhibits negligible performance attenuation (<1%) after bending for 500 cycles, and maintains 96% of its initial performance even after 550 h of indoor exposure. Furthermore, the high-speed OPD demonstrates a high data transmission rate of 80 MHz with a bit error rate of 3.5 $ \times $ 10^−4^, meaning it has great potential in next-generation high-speed flexible optical communication systems.

## Teaser

A flexible high-speed organic photodetector, incorporating a π-extension acceptor in central and end units, exhibits a faster photoresponse than silicon photodetectors, indicating substantial potential in the next-generation optical communication system.

## INTRODUCTION

Optical communication, also called light fidelity (LiFi), is undoubtedly one of the most compelling wireless communication technologies for high-speed and large-capacity data communication without external electromagnetic interference and safety issues [1–4]. Moreover, visible (Vis) infrared (IR) light has frequencies extending from 400 Terahertz (THz) to 800 THz, offering an available frequency spectrum 10 000 times higher than the current radio frequency [5–8]. Thus, LiFi is widely expected to tackle the current bottlenecks in high-speed data transmission that exist in the Internet of Things, light detection and ranging (LiDAR), big data, and consumer electronics [6,9]. Photodetectors (PDs) are core building blocks of LiFi systems, and function as optical receivers that convert optical signals into electrical signals and play a central role in the speed, capacity and accuracy of data transmission [10–14]. Due to the advances in mature design and fabrication expertise, silicon (Si) PDs currently play a dominant role in optical communication and remain unbeatable in terms of performance, infrastructure and practical application [11]. However, achieving high-speed photoresponse generally requires reducing Si device thickness, resulting in a low responsivity due to the weak absorption efficiency of Si, particularly in the near-infrared (NIR) region [9]. More importantly, Si PDs commonly suffer from mechanical rigidity, a small area, high working bias and a fixed bandgap, which are not ideally suited to typical burgeoning fields, particularly wearable electronics, soft robotics, intelligent transport systems (ITSs) and implanted electronics, i.e. those requiring, for example, mechanical flexibility, a large active area, spectrum tunability, low cost and efficient energy consumption [9,11,15–17].

Solution-processed organic photodetectors (OPDs) offer significant mechanical flexibility, a tailorable bandgap and large-area fabrication capability, making them suitable for a wide range of non-standard substrates [17–20]. Moreover, OPDs based on organic semiconductors exhibit a high optical coefficient (>10^5^ cm^−1^), which ensures an ultrathin photoactive film (∼100 nm) for efficient and fast photodetection [20,21]. These attractive characteristics make OPDs promising alternatives for low-cost, flexible and power-efficient information receivers in next-generation integrated and scalable LiFi modules [11]. Benefitting from the rapid development of photovoltaic materials, particularly non-fullerene acceptors (NFAs) with acceptor-donor-acceptor (A-D-A) architectures [22–24], OPDs have facilitated considerable breakthroughs in terms of visible and infrared light communication, even surpassing inorganic photodetectors in some specific metrics [25–27]. Nonetheless, the data transmission speed of OPDs still lags behind their inorganic counterparts due to the relatively slower response speed [19]. The fundamental reason lies in the low charge mobility of organic photoactive films caused by intrinsically disordered molecular packing, leading to high-density trap states and energetic disorders [28–30]. In OPDs with a bulk heterojunction, it is crucial to obtain a suitable nanoscale phase-separated morphology, preferably featuring a three-dimensional (3D) intermolecular penetrating network between donors and acceptors, which can efficiently facilitate charge separation/transfer/transport in the blend film [31]. An outstanding example is Y6 NFA and its derivatives, wherein the central unit is actively involved in intermolecular packing, leading to an effective nanoscale 3D network and improving charge mobility. However, even with these encouraging advancements, the response speed of current OPDs still lags behind inorganic counterparts. Also, the response time obtained via transit photocurrent (TPC) tends to be overestimated as the photocurrent typically fails to reach saturation before switching off irradiation light [32]. Furthermore, the key factors that dominate the response speed of OPDs are seldomly investigated and studied, resulting in a lack of principles guiding high-speed OPD fabrication and optimization, thereby hindering their practical application in flexible LiFi communication systems for high-speed and large-capacity data transmission.

Here, we successfully fabricate a flexible Vis-NIR OPD by introducing a novel NFA (CH17) featuring a remarkable π-extension in the direction of both central and end units with respect to the Y6 series, which forms a more effective and compact 3D molecular packing network. Consequently, the OPD with CH17 exhibits a reduced energetic disorder and lower trap states compared to Y6-based devices, which simultaneously improves charge mobility, decreases noise current and reduces the RC time constant, ultimately leading to a substantial improvement in response speed and sensitivity. The OPD exhibits a significantly fast photoresponse time of 91 ns, low noise current of 3 pA, peak spectral responsivity (SR) of 0.53 A W^−1^ at 830 nm and large linear dynamic range (LDR) of over 130 dB. Notably, under the same measurement conditions, the response speed of CH17-based OPDs surpasses most commercial silicon-based photodiodes and all previously reported NFA-based OPDs. Compared to rigid silicon photodiodes, the flexible NIR OPD demonstrates negligible performance degradation (<1%) after mechanically bending for 500 cycles and maintains >96% of its initial performance after working for 550 h under indoor exposure. Based on this excellent optoelectronic performance, we designed and assembled a LiFi system with flexible OPDs, achieving high-speed data communication of 80 Mbps with a bit-error-rate (BER) of 3.5 $ \times $ 10^−4^, which demonstrates the great potential of OPDs in emerging applications of ITSs.

## RESULTS AND DISCUSSION

### Photoresponse speed of OPDs

**Figure 1.**
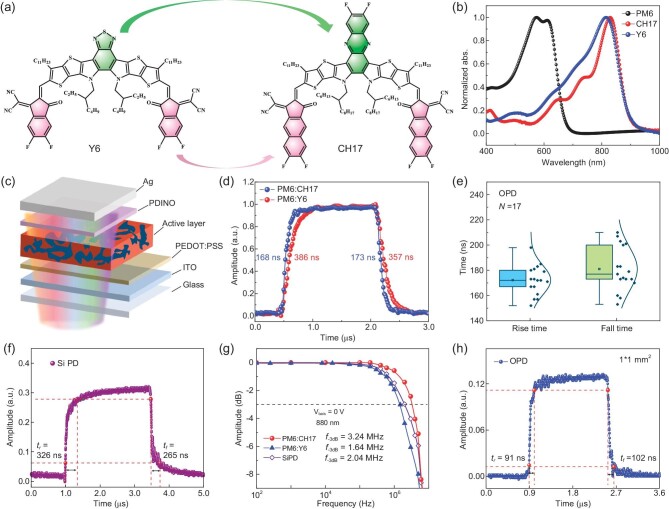
Response speed performance of OPDs and Si PD. (a) Chemical structures of Y6 and CH17 NFAs. (b) Optical absorption profiles of Y6, CH17 and PM6 films. (c) Device structure of OPD based on PM6 : CH17 and PM6 : Y6. (d) Time domain response of Y6 and CH17-based OPDs. (e) Distribution of the response/recover time of the CH17-based OPD under 880 nm illumination. (f) The high-speed Si PD (S1133-01) at zero bias under 880 nm illumination based on steady state analysis. (g) Frequency domain response of the CH17-based OPD, Y6-based OPD and high-speed Si PD (S1133-01). (h) Time domain response of the CH17-based OPD with an area size of 0.01 cm^2^.

Figure 1a illustrates the chemical structures of Y6 and CH17 NFAs. The detailed synthetic process of CH17 is described in Methods according to our previous study [29]. As shown in Fig. 1b, the CH17 film shows a similar absorption profile with an absorption onset of 914 nm. Compared to the Y6 film, the CH17 solid film demonstrates a distinct shoulder peak located at 750 nm, implying a more effective intermolecular π−π packing in the CH17 film. To improve the response speed of OPD, here, a layer of high-mobility poly(3,4-ethylenedioxythiophene) : poly(4-styrenesulfonate) (PSS : PEDOT) is spin-coated on the indium tin oxide (ITO) substrate serving as a hole transporting layer (HTL), concurrently reducing the surface roughness of the substrate, whereas N,N′-Bis (N,N-dimethylpropan-1-amine oxide) perylene-3,4,9,10-tetracarboxylic diimide (PDINO) film works as an electron transporting layer (ETL) and blocks holes owing to the proper energy level shown in Fig. 1c [33]. The energetic levels of photoactive materials, HTL, ETL and electrodes are presented in Fig. S1. The detailed processes of device fabrication and optimization are all described in Methods.

Given that the response speed of OPD plays the most crucial role in determining the data transmission speed, capacity and accuracy in the LiFi system, it is crucial to accurately examine the performance, in terms of speed, of OPDs. We introduced a steady-state current measurement in the dark and under NIR illumination (λ = 880 nm) instead of the commonly reported TPC measurement [34], effectively mitigating the overestimation of response speed performance. Details of the steady-state analysis method of characterizing the response time of OPDs and Si PDs can be seen in Supplementary Note 1 and Fig. S2. As shown in Fig. 1d, the optimal CH17-based OPD (0.04 cm^2^) achieves an impressive response time of 168 ns and a fall time of 173 ns at zero bias, exceeding those of Y6-based OPDs with a corresponding value of 386 ns and 357 ns, respectively (Fig. 1d). Figure 1e displays the distribution of response/recover time across 17 independent CH17-based OPDs, demonstrating consistently reproducible high-speed performance. It is worth noting that the response/recover speed of CH17-based OPDs even suppresses the popular commercial Si PD with a response/recover time of 326 and 265 ns (S1133-01, 0.0672 cm^2^), shown in Fig. 1f. Additionally, when the area size of a CH17-based OPD is increased to the same as that of the Si PD, the CH17-based OPD demonstrates a significantly faster photoresponse (212 ns) than the Si PD (326 ns, shown in Fig. S3). Theoretically, the data transmission speed and capacity are determined by the −3 dB cut-off frequency of OPD, estimated using the equation $f = \frac{{0.35}}{t}$, where *t* is the transit time of OPD [25]. Here, we adopt a modulated optical signal at a wavelength of 880 nm to measure cut-off frequency with a proper filtering process [35], effectively avoiding overestimation or underestimation of the frequency measurement (Supplementary Note 2, Figs S4 and S5). As shown in Fig. 1g, the Y6-OPD, CH17-OPD and Si PD, operating at zero bias, exhibit −3 dB cut-off frequencies of 1.64, 3.24 and 2.04 M Hz, respectively, which is consistent with the measured response time. Moreover, the CH17-based OPD has a constant cut-off frequency over the entire spectrum region and under various illumination intensities, demonstrating considerable potential in high-speed optical communication across the Vis-NIR region (Figs S6a and S7). To further improve the speed performance of OPD, we reduce the pixel size of OPD to 0.1 $ \times $ 0.1 cm^2^, which produces a faster response time of 91 ns with a corresponding −3 dB cut-off frequency of over 4 MHz (Fig. 1h). This exceeds the performance of most commercial Si PDs and reported OPDs with a detection range of over 850 nm, as summarized in Fig. S6b and Table S1.

### Optoelectronic performance of OPDs

Dark current and rectification ratio (RR) are critical metrics of OPDs as they directly determine the specific detectivity (D*), LDR, noise equivalent power (NEP) and rectifying characters of photodetectors [36]. Responsivity (*R*) is a key figure of merit used to evaluate the sensitivity of OPDs, which can be calculated from external quantum efficiency (EQE) values using the following equation:


(1) \begin{equation*}R = \frac{{EQE}}{{100\% }} \times \frac{{{\lambda }_{input}}}{{1240\left( {nm\ W\ {A}^{ - 1}} \right)}},
\end{equation*}

where ${\lambda }_{input}$ is the incident light wavelength.

The specific detectivity (D*) is another critical parameter for assessing the sensitivity of OPDs for ultraweak optical detection, quoted in cm Hz^1/2^ W^−1^, as described by the following equation:


(2) \begin{equation*}{D}^* = \frac{{R\sqrt {AB} }}{{{i}_n}},
\end{equation*}

where *R, i_n_, B* and *A* are responsivity, noise current, bandwidth and effective size of a photodetector, respectively.

The NEP is defined as the incident light power at which the signal-to-noise ratio (SNR) is unity. It can be calculated using the following equation:


(3) \begin{equation*}NEP = \ \frac{{{i}_n}}{R}.
\end{equation*}

**Figure 2.**
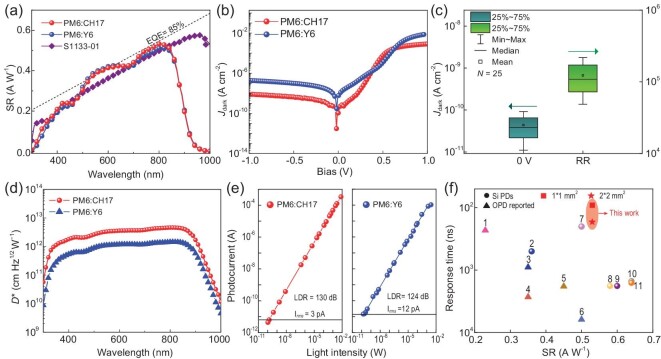
Sensitivity of OPDs and a commercial high-speed Si PD (S1133-01). (a) Spectral responsivity profiles of Y6- and CH17-based OPDs and a commercial high-responsivity Si PD (S1133-01). (b) Current density-voltage (*J*-V) curves of Y6- and CH17-based OPDs operating in the dark. (c) Variation of dark current and RR of 25 independent CH17-based OPDs. (d) Specific detectivity of Y6- and CH17-based OPDs at wavelengths ranging from 300 to 1000 nm. (e) LDR of Y6- and CH17-based OPDs under the irradiation of NIR light (λ=880 nm) at zero bias. (f) Summarization of high-speed OPDs and Si PDs, with numbers 1–11 relating to high-speed OPDs operating without external bias (Table S1).

Owing to the comparable absorption profiles and energetic levels, both Y6 and CH17-based OPDs exhibit similar responsivity profiles over the spectrum from 400 to 900 nm, with a peak spectral responsivity of 0.53 A W^−1^ at 830 nm. Remarkably, the CH17-based OPD shows a superior responsivity compared to the commercially available high-responsivity Si PDs (S1133-01) within the spectrum of 830 nm (Fig. 2a). Compared to the Y6-based OPD, the CH17-based OPD demonstrates a lower dark current both at zero and reverse bias voltage, shown in Fig. 2b. Moreover, the solution-processed CH17 OPDs exhibit exceptional reproductivity with a slight variation of dark current and RR, with a median of 41 pA/cm^2^ and 10^5^ for 25 devices, respectively (Fig. 2c). Unless specifically stated, all measurements of dark current for OPDs were obtained at zero bias to minimize noise current.

It is important to note that the values of D* have often been overestimated in previous literature [37], as the noise current containing thermal noise, shot noise and flicker noise is underestimated and the responsivity is overestimated at low-intensity light [37]. Here, we quantified the sensitivity of OPD by directly measuring the noise current instead of carrying out a calculation [26,37]. The resulting CH17-based OPD exhibits a low noise current of 3 pA (Fig. S8). Benefitting from a lower noise current than the OPD based on Y6, the CH17-based OPD shows remarkable NEP and D* of 4 $ \times $ 10^−11^ W and over 10^12 ^cm·Hz^1/2^W^−1^ (350–900 nm), respectively, which is comparable to commercial Si PDs (S1133-01), shown in Fig. 2d. The LDR is determined by measuring the photocurrent under a series of light intensities, and is extracted by $LDR = 20{\mathrm{log}}( {{\raise0.7ex\hbox{${{J}_{max}}$} \!\mathord{/ {\vphantom {{{J}_{max}} {{J}_{min}}}}} \!\lower0.7ex\hbox{${{J}_{min}}$}}} )$. As shown in Fig. 2e and Fig. S9, the CH17-based OPD exhibits a linear response covering almost eight orders of magnitude under illumination of 880 nm, yielding a significantly large LDR of over 130 dB owing to the low dark current of OPD, which is quite close to the value of 140 dB for commercial Si PDs (S1133-01, Fig. S9) and exceeds the Y6-based OPD. Overall, the CH17-based OPD exhibits an outstanding comprehensive performance regarding response speed and responsivity, as summarized in Fig. 2f.

### Mechanism of high-performance OPD

Accordingly, the response bandwidth of the p-i-n (PIN) photodiode heavily depends on both the resistor-capacitor (RC) time constant and the charge-carrier transit time (*t*) consisting of drift time and diffusion time, shown as follows [38]:


(4) \begin{equation*}{\tau }_r = \sqrt {\tau _{RC}^2 + \tau _{drift}^2 + \tau _{diff}^2},
\end{equation*}

(5) \begin{equation*}{\tau }_{RC} = 2.2RC,
\end{equation*}

(6) \begin{equation*}{\tau }_{drift} = \frac{{{d}^2}}{{\mu V}},
\end{equation*}

where ${\tau }_{RC}$ is the time constant caused by the total series resistance (R_s_) and the sum of the capacitance of device (C), ${\tau }_{drift}$ is the drift time in the depletion region and ${\tau }_{diff}$ is the diffusion time of carriers to the non-depletion region. Generally, the diffusion time of carriers is negligible in the PIN photodiode as the i region is fully depleted [38]. To explore the underlying factors that determine the response speed of OPD, we investigate the response time of OPDs by modulating the RC time constant and charge transit time via tunning device capacitance, series resistance, charge mobility and charge drift length.

**Figure 3.**
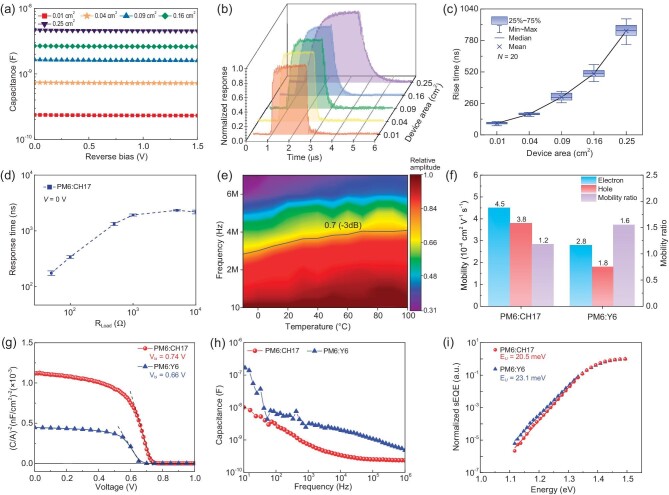
Electric characterizations of OPDs. (a) The capacitance of CH17-based OPDs with different sizes under various reverse biases. (b) Time domain response of the CH17-based OPD with different sizes. (c) The box chart of rise times of OPDs with different device areas. (d) The response time of the CH17-based OPD (0.04 cm^2^) integrating with various external resistances operating at zero bias under 880 nm illumination. (e) −3 dB cut-off frequency of the CH17-based OPD operating at different temperatures ranging from −10 to 100°C at zero bias under 880 nm illumination. (f) Charge mobility of Y6- and CH17-based OPDs. (g) Mott–Schottky plots (dashed lines represent the linear fitting) of Y6- and CH17-based OPDs. (h) Capacitance of Y6- and CH17-based OPDs. (i) Sensitive external quantum efficiency (sEQE) spectra analysis of the energetic disorder of optimized Y6- and CH17-based OPDs at the absorption onset.

As depicted in Fig. 3a, the geometrical capacitance of OPD is directly proportional to the effective area size of the device, which decreases with the reducing area of the device. An analysis of the response time versus pixel area shown in Fig. 3b and c reveals that the response time decreases with decreasing pixel size and does not plateau even at the size of 0.01 cm^2^, indicating that the geometrical capacitance of OPD plays a critical role in determining the response speed. Although the response speed shows a strong dependence on the area, the responsivity of OPDs with different effective areas remains constant, suggesting that the effective area does not affect the sensitivity of OPDs (Fig. S10). Another key factor in determining the RC time constant is the series resistance of the device. As shown in Fig. 3d, integrating increasing external resistors with the CH17-OPD leads to an expected lengthening of the response time, which further confirms the above hypothesis that the response speed of OPD is significantly affected by the RC time constant. The Si PD shows an increasing trend similar to the OPDs shown in Fig. S11. As presented in Fig. 3a and Fig. S12, CH17-based OPD and Si PD capacitances are 0.7 and 1.2 nF, respectively. Consequently, when integrating with the external resistance (*R*) of 50 Ω, the corresponding RC time of the CH17-based OPD and Si PD are ∼81 and 132 ns, respectively. This surprisingly low capacitance of the OPD should be due to the low dielectric constant of organic semiconductors.

According to Equation (4), the corresponding drift times of CH17-based OPDs and Si PDs are 147 and 362 ns, respectively, primarily determined by the thickness of photoactive films and charge mobilities of the photodetectors. By integrating the charge mobility (∼4 $ \times $ 10^−4^ cm^2^V^−1^s^−1^) and thickness (95 nm) of the CH17-based OPD, the drift time of the CH17-based OPD is ∼200 ns obtained via Equation (6), which is quite close to the above-calculated result (147 ns). This significantly short drift time should be due to the ultrathin photoactive film and relatively high mobility of CH17-based OPDs. Despite Si exhibiting a theoretically high charge mobility, the doping process should largely degrade the charge mobility due to the scattering effect. Moreover, Si generally demonstrates a low absorption efficiency (∼10^3^ cm^−1^) in the NIR region, which demands a thick film (>10 μm) to efficiently absorb the incident light. Therefore, the reduced charge mobility, high capacitance and thick photoactive film should lead to the slow photoresponse of Si PDs.

As discussed above, the drift time primarily relies on the charge velocity and drift length, where the charge mobility in organic semiconductors, according to the Gaussian disorder model, is heavily influenced by the working temperature [39]. Generally, charge transport proceeds via hopping in the disordered system; therefore, higher temperatures could facilitate transport by providing the necessary energy to overcome the barriers caused by energetic disorder [39]. As shown in Fig. S13, the electron charge mobility demonstrates an expected increase from 2 $ \times $ 10^−4^ cm^2^V^−1^s^−1^ (263 K) to 1 $ \times $ 10^−3^ cm^2^V^−1^s^−1^ (373 K), leading to a significant increase in the corresponding −3 dB cut-off frequency (Fig. 3e). Remarkably, the −3 dB cut-off frequency of CH17-based OPDs is as high as 4 MHz at 373 K (Fig. S14). This outstanding response speed performance at high temperatures also ensures the suitability of OPDs for high-speed optical communication in harsh environments [39]. Under high reverse bias, the OPD demonstrates a constant response time, owing to the saturation of charge velocity and negligible capacitance change in OPDs under reverse bias (Fig. S15 and Fig. 3a). The thickness of the photoactive film is another critical factor that determines ${\tau }_{drift}$. Benefitting from the high optical absorption coefficient of photoactive materials, together with the large space charge length (107 nm) (Fig. S16), the optimal device with a thickness of 95 nm exhibits a fast response time of 168 ns and high peak responsivity of 0.53 A W^−1^, simultaneously (Table S2). The thicker device has a longer charge transit length, whereas the thinner device exhibits a weaker optical absorption efficiency, resulting in a slower response or lower responsivity (Fig. S17 and Table S2).

As shown in Fig. 3a and Fig. 3f, the CH17-based OPD exhibits a lower capacitance (0.7 nF at 10 KHz), higher charge mobility (4.5 $ \times $ 10^−4^ cm^2^V^−1^s^−1^) and more balanced charge mobilities (e/h = 1.2) than the OPD based on Y6 NFA (1.9 nF, 4.5 $ \times $ 10^−4^ cm^2^V^−1^s^−1^, and e/h = 1.6) according to the space-charge-limited current (SCLC) method (Fig. S18), contributing to a higher response speed. Trap states in organic photodetectors, arising from the disordered nature, structural defects and impurities within the blend film, inherently influence the charge transport and charge storage capability of OPDs, leading to high parasitic capacitance and limited charge mobility [40].

To figure out the underlying mechanism with regard to the impressive performance of CH17-based OPDs, we conducted a capacitance-voltage (C-V) measurement and Mott-Schottky analysis to extract the trap density by the formula ${N}_t = - \frac{2}{{q{\varepsilon }_0{\varepsilon }_r}}{( {\frac{{d{{( {C/A} )}}^2}}{{dV}}} )}^{ - 1}$, where *ε*_r_ is the relative dielectric constant of the photoactive film (assuming *ε*_r_ = 3.5), *ε*_0_ is the vacuum permittivity and *A* is device area [41]. As shown in Fig. 3g, the resulting trap density in the OPD based on CH17 is as low as 7.12 $ \times $ 10^15^ cm^−3^, significantly lower than that of the Y6-based OPD (1.39 $ \times $ 10^16^ cm^−3^). To further investigate the trap density of states (DOS) distribution of CH17- and Y6-based OPDs, we conducted a capacitance-frequency ($\omega $) measurement as illustrated in Fig. 3h. The DOS distribution can be extracted using the following equation:


(7) \begin{equation*}DOS\!\left( {{E}_\omega } \right) = - \frac{{{V}_{bi}}}{{qAW}}\frac{\omega }{{kT}}\frac{{dC}}{{d\omega }}.
\end{equation*}

As depicted in Fig. S19, the OPD based on CH17 exhibits a reduced sub-band DOS trap state compared to the OPD based on Y6 across the trap energy range from 0.35 to 0.55 eV, which is consistent with the improved photoresponse speed and reduced dark current in CH17-based OPDs. The disorder is another crucial factor that dominates the charge mobility of OPDs [42]. To quantify the energetic disorder of the blend film, we introduced Urbach Energy (*E_U_*) to describe the width of the tails of the electronic DOS. Remarkably, the CH17-based OPD exhibits a considerably lower *E_U_* value (20.5 meV) than the Y6-based device (23.1 meV) (Fig. 3i), effectively minimizing charge recombination and improving charge transport. This promising result suggests that extending the central and end units could reduce the energetic disorder and improve charge mobility.

Previous studies have indicated that various factors, including energetic disorder, trap states and energetic levels of donors and acceptors, primarily dominate the dark current in OPDs operating in photovoltaic mode [42,43]. Organic semiconductors, in particular, exhibit a broad distribution of energy states extending into the bandgap, resulting in the formation of band tail states occupied by a relatively low concentration of thermally excited carriers that are spatially far from the band edges. Consequently, the effective barriers to thermally excited charges decrease as the energy state distribution increases, increasing free charges and high dark current. Based on the Shockley-Read-Hall (SRH) theory, the thermally excited electron jumps from the donor highest occupied molecular orbital (HOMO) to a trap state located in the middle of the bandgap, which is followed by a second excitation and further release to the lowest unoccupied molecular orbital (LUMO) of the acceptor [40]. This trapping-detrapping process contributes to the thermally excited free charges until equilibrium is attained. Given that the thermally excited charges linearly increase with the number of trap states, a higher concentration of trap states generally results in a higher dark noise current. As shown in Fig. S1, the CH17 shows a similar LUMO level (−3.89 eV) to the Y6 NFA (−3.87), forming similar electron barriers between the HTL and acceptors in OPDs with CH17 and Y6. Therefore, the CH17-based OPD shows a relatively lower dark current than OPDs based on Y6 acceptors due to the reduced energetic disorder and trap states, leading to high detectivity.

Intrinsically, all these electric properties are correlated to the molecular packing and interactions within the blend film. According to our recent study, CH17 NFA, featuring a π−extension in the direction of both central and end units, shows a highly compact 3D packing network. Specifically, the single-crystal CH17 forms a smaller square-shaped void with a side length of ∼12.8 Å compared to Y6 with a rectangle-shaped void of ∼29.2 × 22.2 Å. In the single-crystal X-ray shown in Fig. S20, CH17 exhibits four similar packing modes to Y6, including Mode 1 and Mode 2 (end unit to end unit (‘E/E’ mode)), Mode 3 (end unit to bridged thiophene (‘dual E/b’ mode)) and Mode 4 (end/end unit and central/central unit (‘E/E + C/C’)). Notably, the CH17 shows a more compact packing in Modes 2, 3 and 4, which shows a shorter π−π packing distance compared to the Y6 NFA. Owing to the multiple conjugation extension in both central and end units, the CH17 has a significantly larger facial overlap between adjacent packing molecules in Mode 4 consisting of both ‘E/E’ and ‘C/C’ packing, leading to an enhanced molecular interaction. In addition to these four packing Modes, the CH17 possesses a new packing Mode where the end unit of one molecular almost completely stacks on the central unit of another molecule. This particular packing Mode has a smaller π–π packing distance of 3.36 Å compared to Mode 4 of Y6 with a distance of 3.37–3.40 Å [33]. Such efficient and compact 3D molecular packing should be contributed to the multiple conjugation extension in both end and central units of CH17, which could decrease energetic disorder and trap states, thereby leading to high charge mobility and low capacitance.

### Optoelectronic performance of flexible OPDs

**Figure 4.**
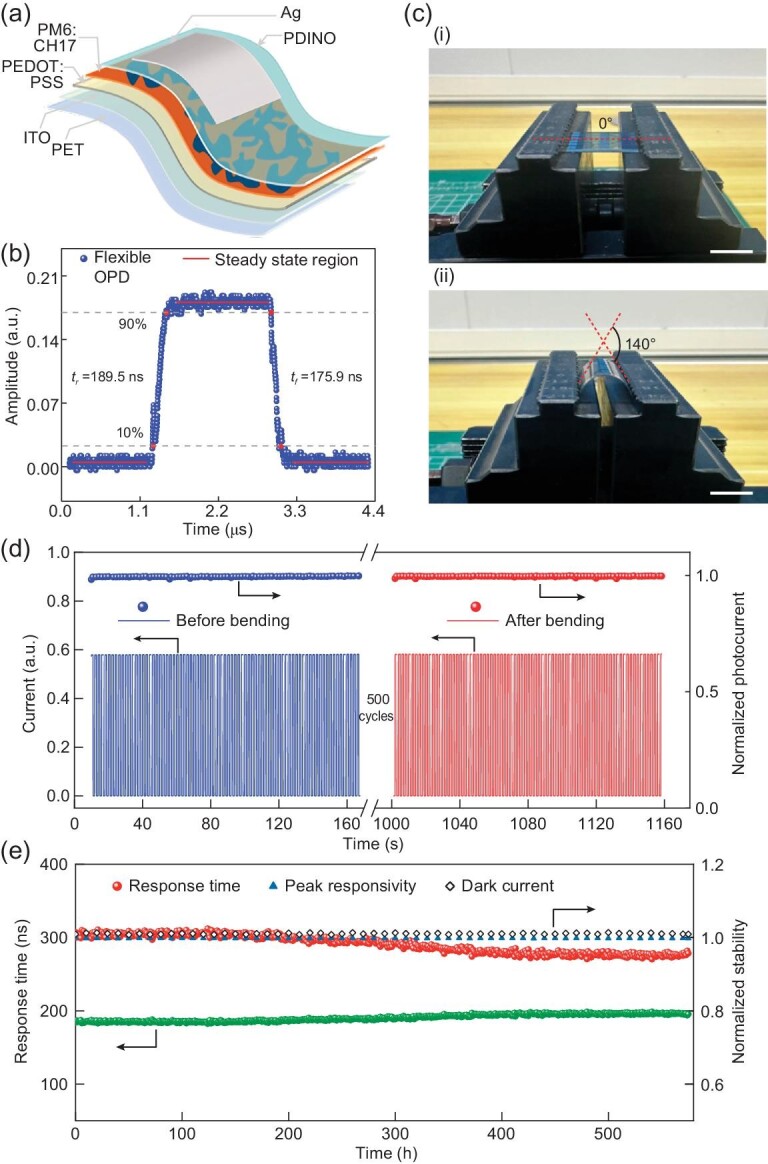
Optoelectronic performance of flexible OPDs. (a) Schematic of the flexible OPD prepared using ITO/PET substrates with an ITO thickness of 30 nm. (b) Time domain response of the flexible OPD at zero bias under 880 nm illumination. (c) Optical graph of mechanical durability test set-up (device area : 0.04 cm^2^, scale bar : 1 cm). (d) Photoresponse of the flexible OPD before and after bending for 500 cycles (irradiation pulse frequency : 0.5 Hz, zero bias, 880 nm). (e) Optoelectronic performance of the flexible device under indoor exposure.

Flexible high-speed OPDs simultaneously targeting mechanical stability and fast response are a prerequisite to achieving high-speed optical communication in wearable electronics. Taking advantage of the exceptional mechanical flexibility of organic semiconductors, we prepared flexible high-speed OPDs that simultaneously achieved a high cut-off frequency and outstanding mechanical stability for optical communication. Figure 4a displays a schematic of the device structure and materials of a flexible OPD, where the indium tin oxide/polyethylene terephthalate (ITO/PET) film works as a flexible substrate due to its high conductivity, excellent flexible durability and high transparency. By optimizing the fabrication process of device and the thickness of ITO electrodes (Fig. S21), an optimal flexible OPD with an ITO thickness of 30 nm gains an extremely fast response time of 189.5 ns and fall time of 175.9 ns, as shown in Fig. 4b. It also has a −3 dB cut-off frequency of ∼3 MHz and high peak responsivity of 0.47 A W^−1^ (Fig. S22). Its performance is quite close to the corresponding rigid OPD, and exceeds the response speed of commercial rigid Si PDs. To the best our knowledge, this is the fastest flexible OPD ever reported (Table S3).

Despite the outstanding performance of OPDs, the path to their commercialization has been impeded by their limited mechanical durability and electrical stability. To assess the commercial aspect of flexible OPDs, we examined the electrical stability of flexible OPDs under severe mechanical bending, ensuring continuous optical communication under conditions of mechanical movement. Figure 4c displays an optical photograph of the mechanical durability test under bending, where the flexible OPD is mounted on a homemade durability tester machine controlled by a microcontroller. After bending for 500 cycles with a bending angle ranging from 0º to 140º, the flexible OPD demonstrates a negligible degradation in photocurrent (<1%) and response speed (Fig. 4d and Fig. S23). Moreover, our device maintains over 96% of its initial response speed, photocurrent and dark current after exposure indoors for 550 h, including a temperature of 25°C and a humidity of 40%. This remarkable mechanical durability and electrical stability present its compelling advantages over the Si PD and ensure its potential application in wearable optical communication systems.

### Optical communication performance of flexible OPDs

**Figure 5.**
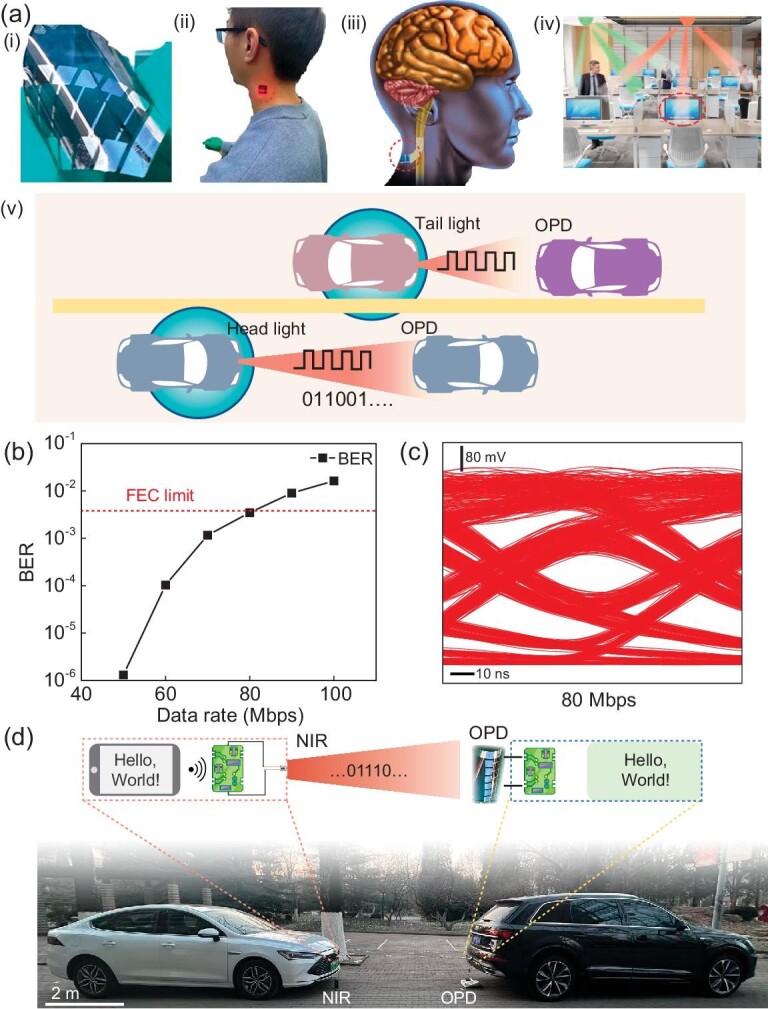
Optical communication of the flexible OPD. (a) Application scenarios of optical communication based on the flexible OPD : (i) Optical graph of the flexible OPD, (ii) flexible OPD attached on skin, (iii) flexible OPD implanted under skin, (iv) OPD used in indoor communication, (v) OPD for intelligent traffic system. (b) The BER of OPD application in high-speed communication. (c) Eye diagram at a communication rate of 80 Mbps. (d) Schematic of the infrared light communication used in vehicle interconnection based on an OPD in infrared light.

To demonstrate the feasibility of the flexible NIR OPD being used in realistic NIR optical communication, we integrated the OPD as an optical signal receiver into the optical communication system for wireless data transmission at a wavelength of 880 nm. Figure 5a shows the potential applications of flexible OPDs in optical communication systems, such as wearable electronics, implanted devices, indoor optical communication and ITSs. We first evaluated the data transmission performance by measuring the BER using a non-return to zero (NRZ) modulation scheme. Generally, the BER should be <3.8 $ \times $ 10^−3^ to achieve reliable optical communication, which is under 7% pre-forward-error-correction (pre-FEC) [44]. As shown in Fig. 5b, our device demonstrates a maximum achievable data rate of 80 Mbps with a BER of 3.5 $ \times $ 10^−4^. To visualize the BER of the OPD at different transmission rates, we also performed an eye-diagram measurement with an intrinsic rate of 10 and 80 Mbps, which showed an open and clear eye diagram, indicating that the flexible OPD can be readily used for practical optical communication (Fig. 5c and Fig. S24).

Optical communication has superior advantages when applied to ITSs than current radio frequency wireless technology, particularly in achieving high-speed and efficient vehicle communication via tail lights or near-infrared lights for emergency braking warnings, intersection collision warnings and emergency calls in appalling weather [45,46]. Taking full advantage of our OPD with regard to high-speed response and high responsivity, we designed a text communication system using an OPD, which combined modern radio communication technology with optical communication, achieving long-distance optical communication between two cars via NIR light. For example, a mobile phone sends the command ‘Hello, World!’ to a microcontroller that can modulate an NIR LED to send an optical signal, as presented in Fig. 5d. Then the OPD receives the optical signal emitted from the NIR LED and converts it into the text ‘Hello, World!’ (Supplementary Video 1 and 2). This encouraging result directly proves that our flexible high-speed OPDs have great potential in future high-speed optical communication systems from the visible to NIR range.

## CONCLUSION

In conclusion, we have successfully fabricated a flexible high-speed OPD by introducing a novel NFA with a more compact 3D molecular packing network, enabling performance, in terms of speed, to be significantly improved beyond that of previously reported OPDs and most commercial Si PDs in self-powered mode. The OPD achieved a response time of 91 ns, −3 dB bandwidth of 4 MHz and responsivity of 0.53 A W^−1^ at 830 nm. More importantly, the flexible OPD displays outstanding mechanical and electrical stability after mechanical bending 500 times with negligible performance degradation (<1%) and maintained over 95% of its initial photoresponse after indoor exposure for 550 h. All these outstanding results are contributed to by the novel CH17 NFA featuring a prominent π-extension in the direction of both central and end units, forming a favorable compact 3D molecular packing in the blend. This multiple extension in central and end units effectively reduces the packing distance and enhances the intermolecular interaction, efficiently reducing trap states and energetic disorder in the OPD, thereby improving charge mobility and capacitance. In addition, the flexible OPD-based LiFi system for high-speed optical communication demonstrated a high data transmission rate of 80 Mbps with a BER of 3.5 $ \times $ 10^−4^, achieving accurate optical communication between two vehicles. The excellent optoelectronic performance and outstanding mechanical stability of flexible OPDs showcases potential practical applications in next-generation optical communication technologies, particularly in flexible electronics, soft robotics, autopilot and implanted devices.

## METHODS

Polymeric donor PM6 and acceptor Y6 from Solarmer Material (Beijing) Inc. and chloronaphthalene were purchased from Sigma Aldrich. PDINO was from eFlexPV Limited and Seniormaterial. The CH17 NFA was synthesized according to our previously reported method [33], which was determined by mass spectrometry, and ^1^HNMR and ^13^CNMR, shown in Figs S25–S27. All the other reagents and chemicals used in this work were purchased from commercial suppliers and used directly without further purification. Flexible ITO/PI transparent electrodes with various ITO thicknesses of 12, 23, 125, 135 and 185 nm were purchased from Advanced Election Technology CO., Ltd. and Xiangchen Technology.

Details on device fabrication can be found in Supplementary Data. Rigid and flexible OPDs based on active layers of PM6 : CH17 and PM6 : Y6 blends were fabricated with a normal architecture of glass/ITO/PEDOT : PSS (4083)/active layer/PDINO/Ag.

Current density-voltage (*J*-V) in the dark and current-time (I-T) curves under irradiation of 880 nm at a pulse frequency of 0.5 Hz and zero bias were tested using a semiconductor parameter analyzer (B1500A, Keysight) and source meter (Keithley 2400 controlled by the software developed by Wuhan Zeal Young Technology Co., Ltd.). The dependency of responsivity with wavelength was performed on QE-R (Enli Technology Co. Ltd.). The noise current of the devices was measured using an electrometer (6517B, Keysight). The capacitance of devices was measured via an electrochemical workstation (Zennium-E, Zahner). Response time and −3 dB cut-off frequency were measured by a high-resolution oscilloscope (MDO4104C, Tektronix) through a square-wave modulated 880 nm LED controlled by a signal generator (DG990, RIGOL). The rise time and fall time of the 880 nm LED was measured using a high-speed PIN detector (Hamamatsu S1223; see Fig. S28). The effects of bias and resistance on response time were investigated using a DC bias module (PBM42, Thorlab) and a variable resistance BNC terminator (VT2, Thorlab). Device thicknesses were tested by a profilometer (Dektak 150, Bruke). A variable temperature test for cut-off frequency was carried out using a dry thermostat (GC-100, Yooning). The UV-Vis spectrometric absorption of PM6 and CH17 films was measured using a UV-Vis spectrometer (Cary 3000, Agilent). Fourier transform photocurrent spectroscopy (FTPS, Enli Technology Co. Ltd.) was used to determine sensitive external quantum efficiency spectra. The hole and electron mobilities were determined by the SCLC method. Mechanical stability was characterized using a homemade set-up. Long-term stability was carried out by measuring response time every other hour. The BER and eye diagram of the OPD in high-speed communication were simulated using Optisystem software. The flow chart is shown in Fig. S29. An IR light text communication system based on an OPD was composed of transmitter circuitry (Tx) and receiver circuitry (Rx). The circuit detail can be seen in Fig. S30.

## Supplementary Material

nwad311_Supplemental_Files
