# Supplementary material for: High-speed flexible near-infrared organic photodiode for optical communication
Source: Natl Sci Rev. 2023 Dec 11;11(3):nwad311. doi: 10.1093/nsr/nwad311 (PMC10833469; doi:10.1093/nsr/nwad311)
Supplement: nwad311_Supplemental_Files [file nwad311_supplemental_files.zip › Supplementary Data.pdf]

SUPPLEMENTARY INFORMATION

High-Speed Flexible Near-Infrared Organic Photodiode for Optical Communication

Yu Zhu,[1] *Hongbin Chen,*[1] *Ruiman Han,*[1] *Hao Qin,*[1] *Zhaoyang Yao,*[1] *Hang Liu,*[1] *Yanfeng Ma,*[1] *Xiangjian Wan,*[1] *Guanghui Li,*[1 *] *Yongsheng Chen*[1,2 *]

[1] The Centre of Nanoscale Science and Technology and Key Laboratory of Functional Polymer Materials, Institute of Polymer Chemistry, College of Chemistry, Nankai University, Tianjin 300071, China.Renewable Energy Conversion and Storage Center (RECAST), Nankai University, Tianjin 300071, China

[2] State Key Laboratory of Elemento-Organic Chemistry, Nankai University, Tianjin, 300071, China

*Corresponding authors: ghli1127@nankai.edu.cn; yschen99@nankai.edu.cn

Device fabrication

Organic Photodetectors based on active layer PM6:CH17 and PM6:Y6 were fabricated with a normal architecture of glass/ITO/PEDOT:PSS (4083)/active layer/PDINO/Ag. Firstly, the glass/ITO substrate was sequentially pre-cleaned in an ultrasonic bath of detergent, deionized water, acetone and isopropanol, followed by drying using N_2 gas. Then the ITO substrate was irradiated by UV light in an ultraviolet-ozone chamber (Jelight Company) for 15 min. A thin layer of hole transport layer was prepared by spin coating the poly(3,4-ethylene dioxythiophene):poly(styrene sulfonate) (PEDOT:PSS, Baytron PVP Al 4083) solution at 4300 rpm for 20 s on the ITO substrate, followed by baking 150□ for 20 min in air and then transferred to a glove box filled with argon. The PM6:CH17 or PM6:Y6 mixture was fully dissolved in chloroform (CF) at 50□ with 0.5% of 1-chloronaphthalene (CN) as additive at a concentration of 8 mg/mL of PM6. To remove CN existing in the photoactive film, the obtained device was annealed at 110 □ for 10 min on hot plate. Devices with different thickness of active layers are obtained by tuning the spin-coating speed ranging from 500 rpm to 2500 rpm for 30 s, and the optimal speed is 2000 rpm. After that, PDINO (dissolved in methanol with the concentration of 1 mg/mL) solution was spin-coated on the top of the active layer and obtained a layer of electron transporting layer with a thickness of 15 nm. Finally, Ag electrode with the thickness of 100 nm was prepared by thermal evaporation technique under 2×10^{-6} Pa. The active area of the final device was 0.04 cm^2 determined by a predesigned mask. For long-term stability test, device was encapsulated by an epoxy resin (Norland Optical Adhesive 81) under UV of 365 nm exposure for 2 mins. To prepare flexible OPD device, a thin layer of Polydimethylsiloxane (PDMS) film was prepared by spin-coating the mixture of precursors and crosslinkers (10:1) on precleaned glass at 5000 rpm for 30 s, then the unsolidified film was baked at 80 □ for 10 min to get the final PDMS. Then the flexible ITO/PI substrate was attached on PDMS for flexible OPD fabrication. The fabrication process of flexible OPD follows the procedure of rigid device. The optimal flexible device is prepared by spin-coating PM6:CH17 solution at 2000 rpm for 30 s.

Device Characterizations

The space-charge-limited current method was used to measure the hole and electron mobilities, by using a diode configuration of ITO/PEDOT: PSS/active layer/MoO3/Al for hole and ITO/ZnO/active layer/Al for electron. The mobilities were estimated by taking current-voltage curves and fitting the results based on the equation listed below:

$$J = \frac{9\varepsilon_0\varepsilon_r\mu V^2}{8L^3}$$

where J is the current density, ε_0 is the vacuum permittivity, ε_r is the relative dielectric constant, μ is the mobility, and L is the film thickness. V ($=V_{app} - V_{bi}$) is the internal voltage in the device, where V_{app} is the applied voltage to the device and V_{bi} is the built-in voltage due to the relative work function difference of the two electrodes. The long-term stability was carried out by measuring response time every other hour.

Mechanical stability characterizations

Mechanical durability is conducted by bending flexible OPD via a stage transmission, which is controlled by a single-chip microcomputer. The bending angle is controlled by tune the distance of the two stages. The photoelectronic performance was characterized before and after mechanical bending.

Optical communication measurement

Infrared light text communication system based on organic photodetector is composed of transmitter circuitry (Tx) and receiver circuitry (Rx). The circuit detail can be seen in Fig. S30. In order to simulate actual circumstance, we connected the phone with the transmitter via Bluetooth wireless technology to input signals. By texting the desired information in the phone, it can transmit the signal to the single-chip microcomputer and then modulate infrared LED (880 nm). Then, OPD received the infrared light signal and transfer it to electric signal, which was converted to text displaying on Liquid Crystal Display (LCD).

In practical applications, the signal transmitting distance is a key metric to evaluate the performance of optical communication system, which is determined by the responsivity of OPD, irradiation power of LED, and the background environment. Under the low-bias driving (3V) of LED, the infrared light text communication system can perform well and the maximum

communication distance is up to 5 m. Furthermore, we applied this system in vehicle communication by installing the LED and OPD on two cars.

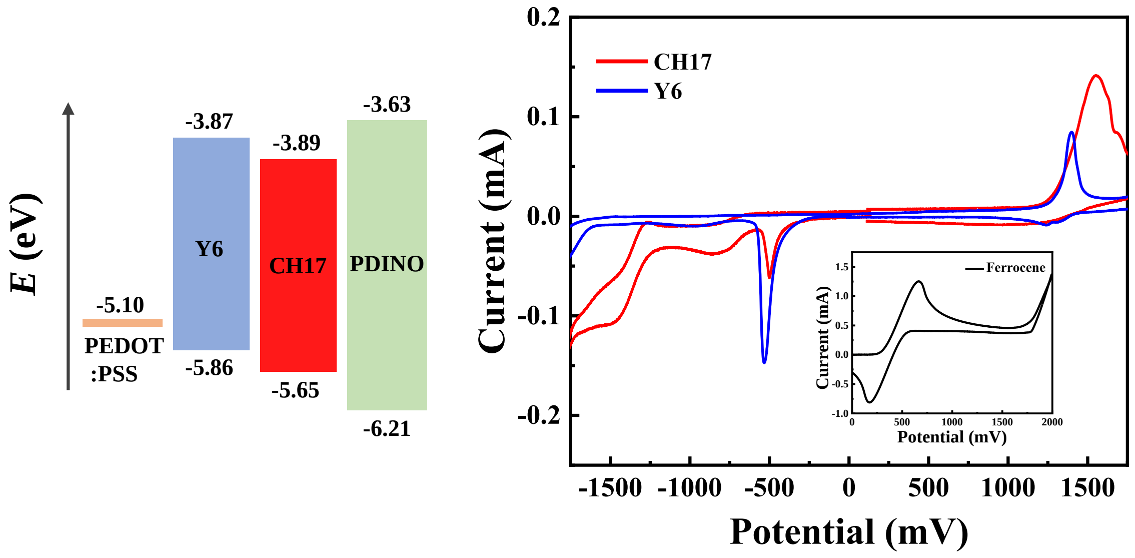

Fig. S1. Energy diagrams of Y6 and CH17 (left). Cyclic voltammograms of the reference (ferrocene) and CH-4Cl and CH-4F films (right). Ferrocene: oxidation and reduction cycle (Inset).

Supplementary Note 1.

Response time was determinate by steady state method, where steady-state is defined as the condition when the average value of $V\ (t_j)$ over N discrete points in time region according to previous reports[1]: $\overline{V(t_j)} = \frac{1}{N} \times \sum_{j=1}^{j=N} V\ (t_j)$, becomes approximately invariant. Mathematically, when the time region:

$$\overline{V(t_j)} - \overline{V\left(t_{j+N}\right)} < V_{rms};$$

where $$V_{rms} = \sqrt{\overline{(V(t_j) - \overline{V(t_j)})^2}} = \sqrt{\frac{1}{N} \times \sum_{j=1}^{j=N}\left[V(t_j) - \overline{V(t_j)}\right]^2};$$

Namely, the difference of average of two adjacent sets $(\overline{V(t_j)} - \overline{V\left(t_{j+N}\right)})$ is less than the standard deviation (V_{rms}). Generally, within 5% of variations is also allowed. $V\ (t_j$ represents voltage amplitude at some time. Averages set typically value between N = 10 to 50 points. In this paper, N=50 is adopted, as shown in **Fig. S1**. When the regions of light and dark steady-state are

determined, response time is measured from time variations corresponding to 10% to 90% (rise time) and 90% to 10% (fall time) of the difference between two steady-states.

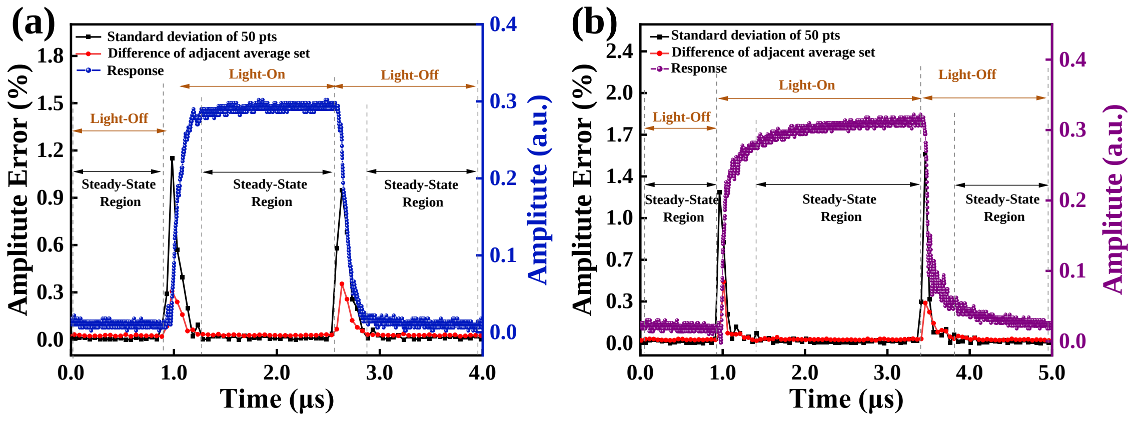

Fig. S2. Response time measurement of PM6:CH17 and Si PD (S1133-01) via steady-state analysis method. (a) Error analysis of optical steady state and dark steady state of OPD. (b) Error analysis of optical steady state and dark steady state of Si PD.

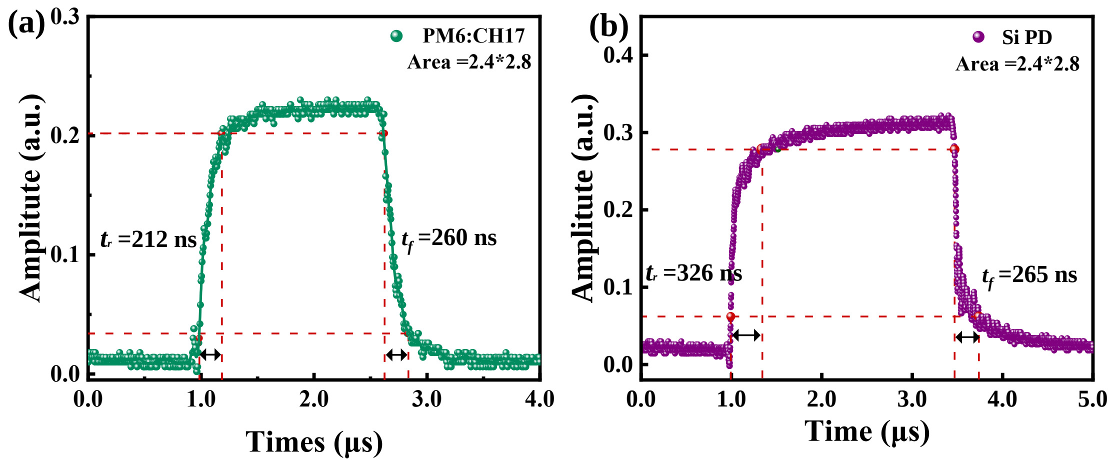

Fig. S3. Photoresponse time of PM6:CH17 (a) and commercial Si PD (b) with 2.4*2.8 mm^2.

Supplementary Note 2.

Cutoff frequency is measured based on steady-state method instead of the calculation from the formula estimation or TPC test. However, it become difficult to accurately capture the amplitude current at high frequency due to the noise generated by oscilloscope. If the signal is not processed by filter, the amplitude is always overestimated, leading to an error in the cutoff

frequency. Although 20 MHz bandwidth is used in photoresponse time test, it obviously is not enough for noise cancellation, as shown in Fig. S1.

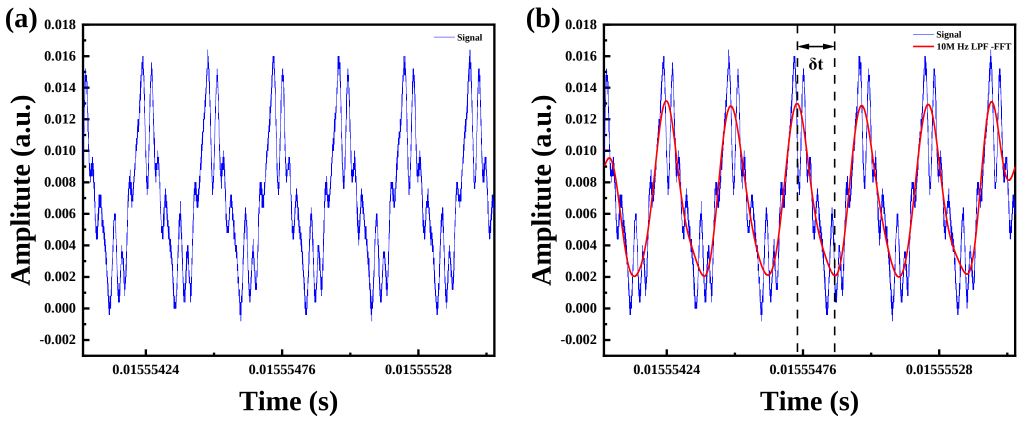

Fig. S4. The original signal obtained in the condition of instrument built-in 20 MHz bandwidth. Therefore, Further data processing is necessary. In this paper, we select 10 MHz low-pass filtering for data processing, which maximize noise cancellation and ensure true amplitude, simultaneously.

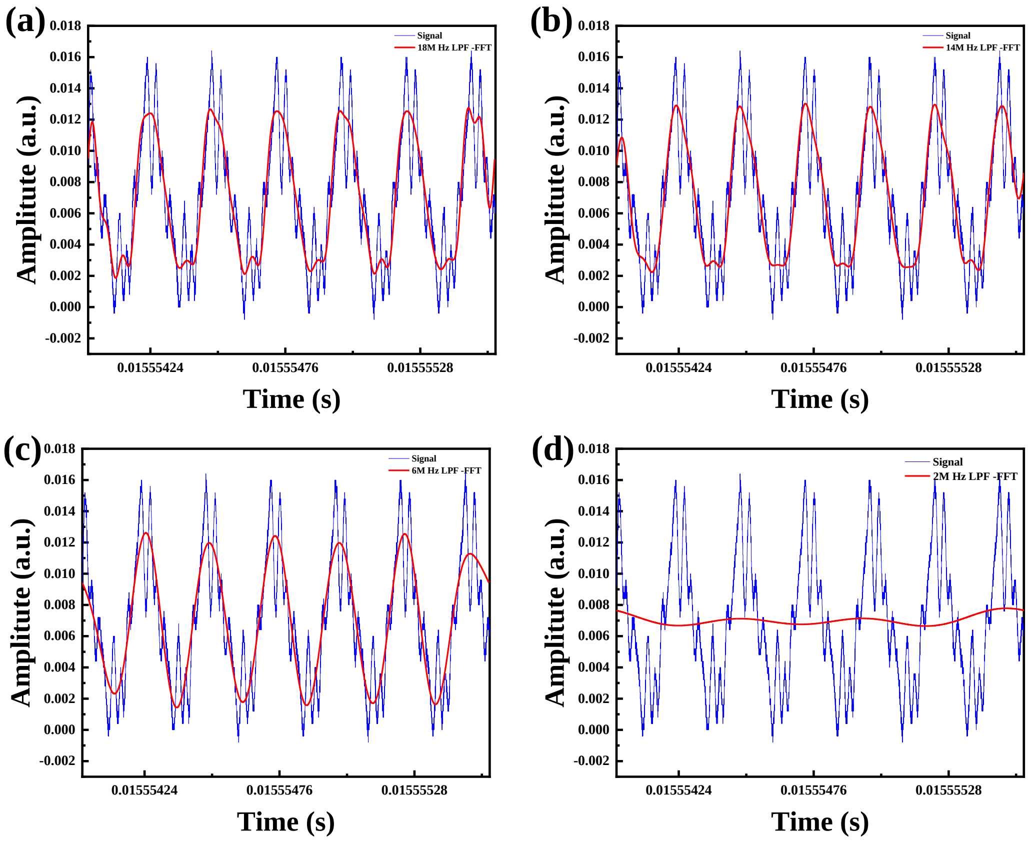

Fig. S5. The original signal (blue line) and proceed signal (red line) after varying degrees of noise

reduction processing

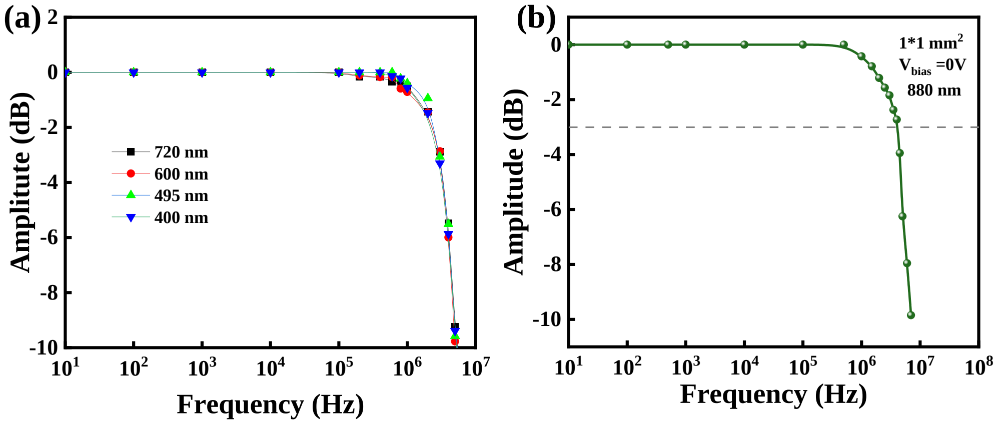

Fig. S6. (a)-3dB cutoff frequency of OPDs under illumination at different wavelength. (b) -3dB cutoff frequency of device with area of 1*1mm^2 that exceeds 4M Hz.

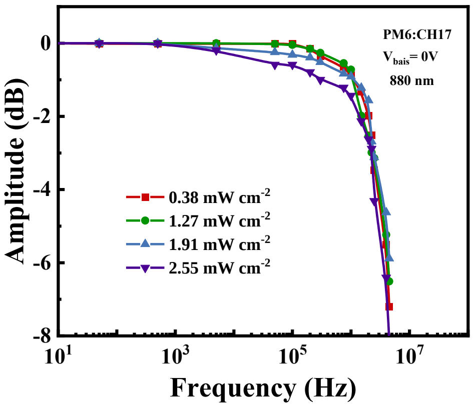

Fig. S7. -3 dB cutoff bandwidth of PM6:CH17 OPD at different light intensities with 880 nm.

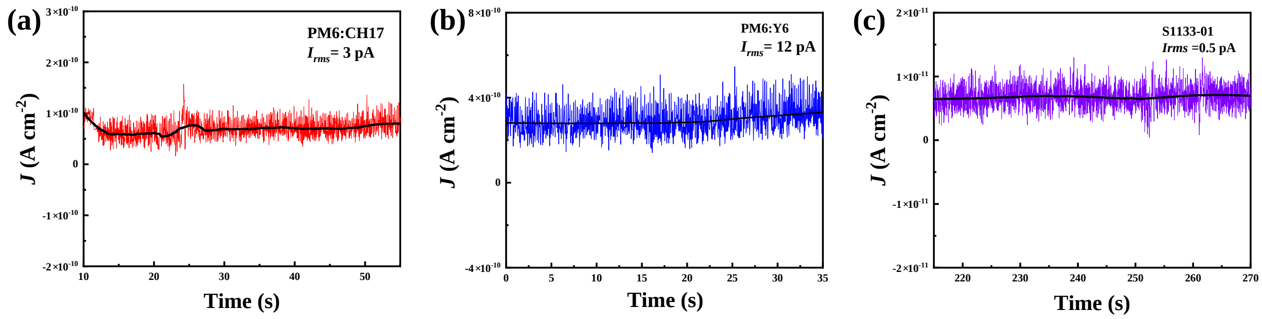

Fig. S8. Noise current of OPD and Si PD. *NEP* and D^* can be calculated from *Irms*[1]. *NEP* of

PM6:CH17, PM6:Y6 and Si PD is 40, 155 and 2 pW, respectively. D^* is obtain by $D^* = \frac{\sqrt{AB}}{NEP}$, Correspondingly, D^* is 2.5×10^{12}, 0.8×10^{12} and 2.2×10^{10} cm·Hz $^{1/2}$ W $^{-1}$ at 880 nm.

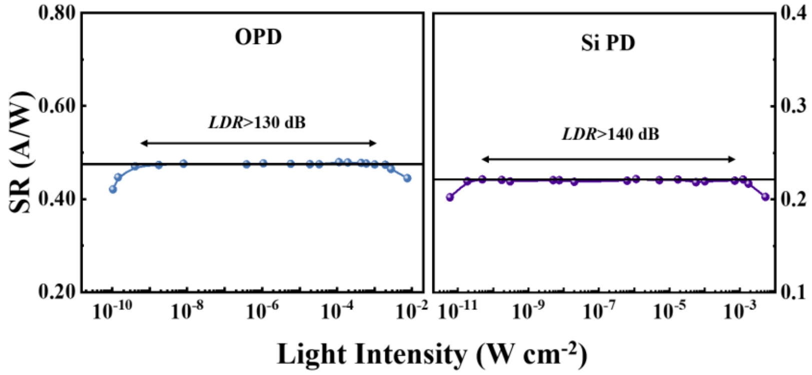

Fig. S9. Linear response range of PM6:CH17 (left) and Si PD (S1133-01) (right).

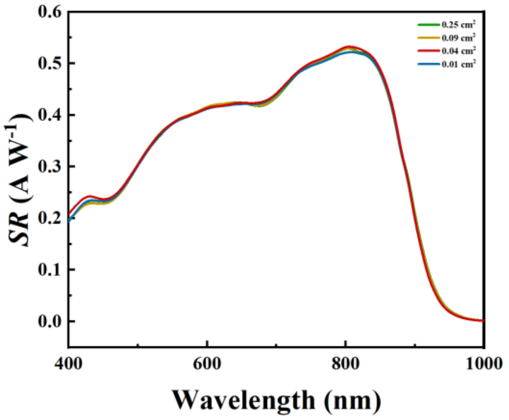

Fig. S10. Responsivity of PM6:CH17 device with of different areas.

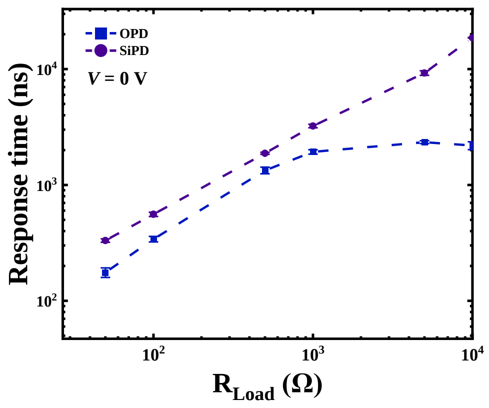

Fig. S11. The dependence of Response time (rise time) on load series resistance

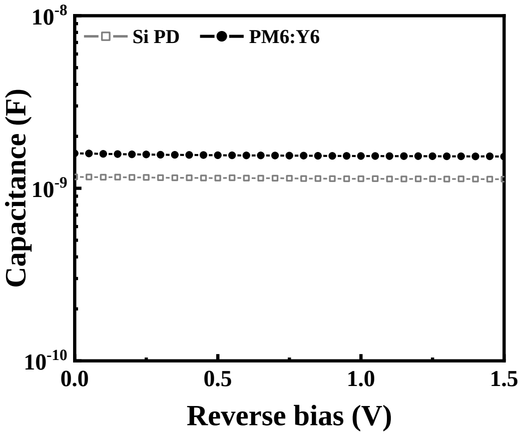

Fig. S12. The capacitance of PM6:Y6 (area: 0.040 cm^2) based OPD and Si PD (area: 0.067 cm^2) under various reverse bias

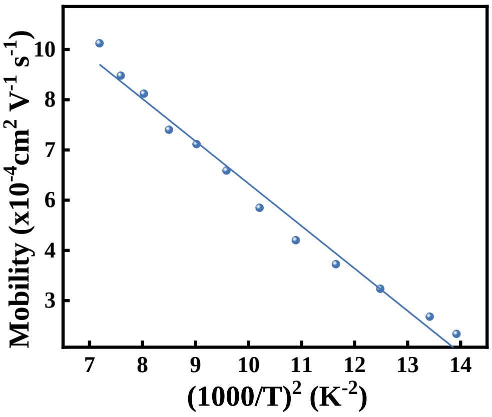

Fig. S13. The dependence of electron mobility on temperature. The energetic disorders of OPD are quantitatively measured based on model of Gaussian distribution of DOS: $\mu = \mu_\infty exp(-(\frac{2\sigma}{3k_BT})^2)$ at zero field. The energetic disorders σ is 41 meV.

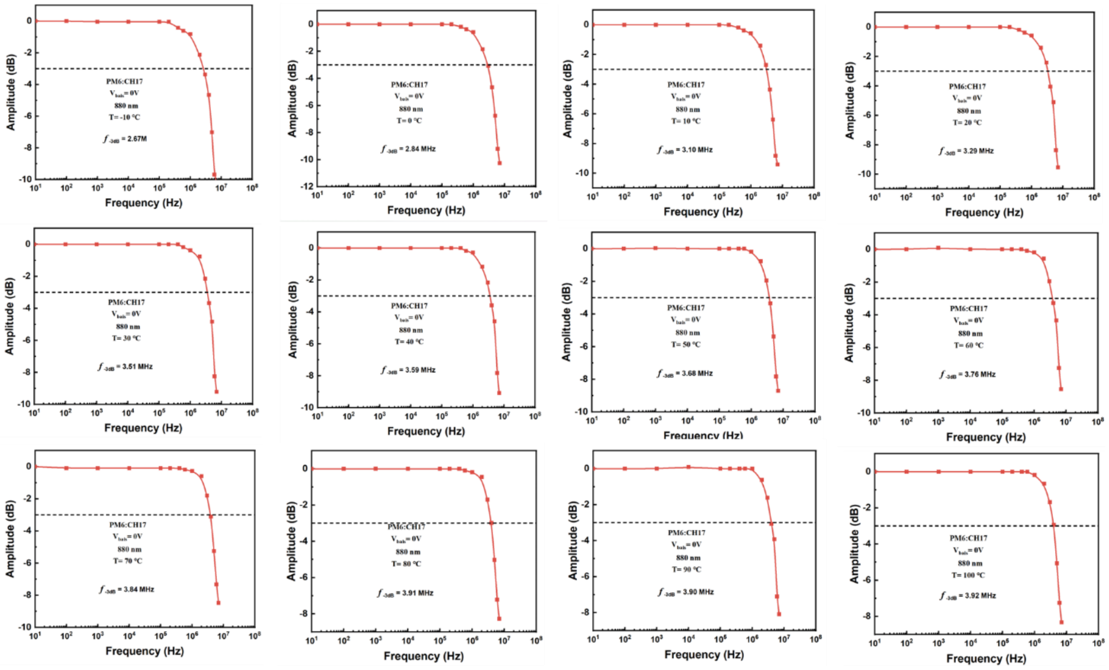

Fig. S14. Temperature dependence of -3dB cutoff frequency from -10□ to 100□.

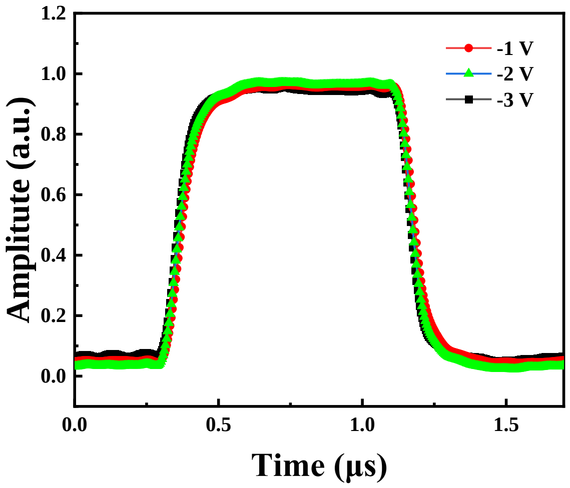

Fig. S15. Photoresponse time of OPD operating at different reverse bias.

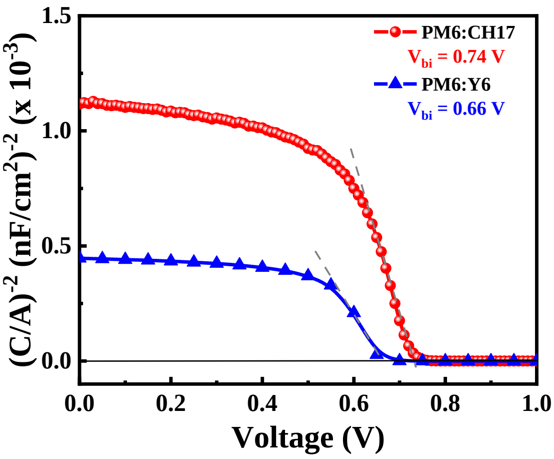

Fig. S16. Mott–Schottky plots of PM6:CH17 and PM6:Y6 devices, measured at 10 kHz in the dark. The depletion layer width is calculated by doping concentration N_t .

$$w = \sqrt{\frac{2\varepsilon_0\varepsilon_r(V_{bi} - V)}{qN_t}}$$

Where $N_t = -\frac{2}{q\varepsilon_0\varepsilon_r}(\frac{d(C/A)^2}{dV})^{-1}$. Doping concentration of PM6:CH17 and PM6:Y6 is calculated as 7.12 $\times 10^{15}$ cm^{-3} and 1.39 $\times 10^{16}$ cm^{-3}, respectively. Correspondingly, the depth of SCR is 107 nm and 72.5 nm.

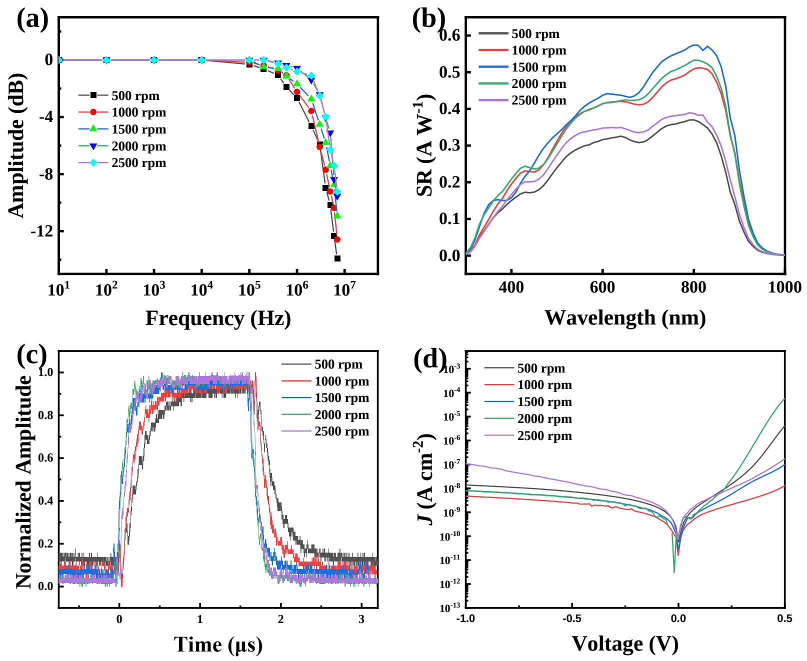

Fig. S17. Performance at different thickness corresponding to different spin speeds. (a) -3dB cutoff frequency of devices with various thickness. (b) responsivity of devices with various thickness. (c) Response time and (d) *J*-V plot.

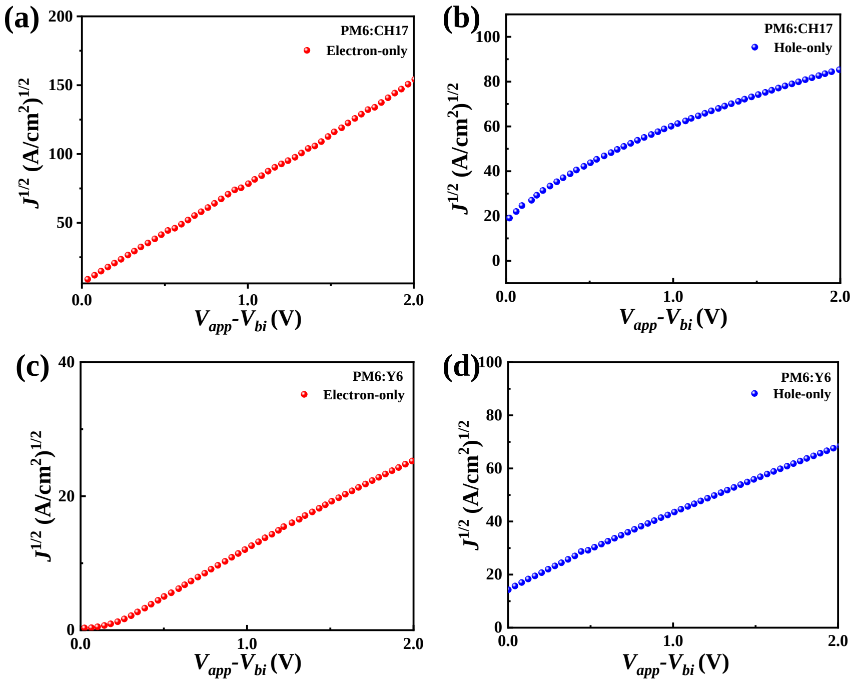

Fig. S18. Measurements of electron and hole mobility. SCLC characteristics of PM6:CH17 and PM6:Y6. (a) 4.5×10^{-4} $cm^2V^{-1}s^{-1}$ electron-only and (b) 2.8×10^{-4} $cm^2V^{-1}s^{-1}$ hole-only of PM6:CH17. (c) 3.8×10^{-4} $cm^2V^{-1}s^{-1}$ electron-only and (d) 1.8×10^{-4} $cm^2V^{-1}s^{-1}$ hole-only of PM6:Y6.

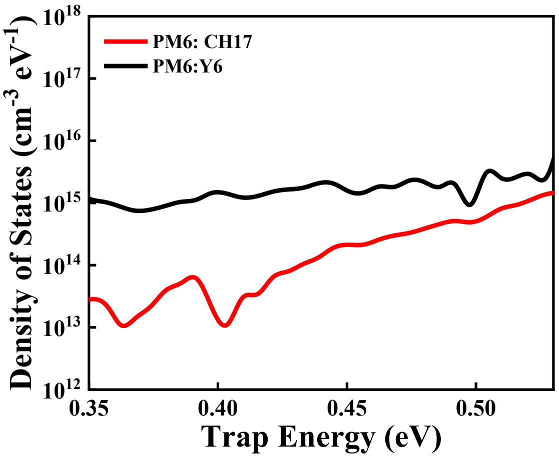

Fig. S19. Sub-band DOSs trap state of PM6:CH17 and PM6:Y6.

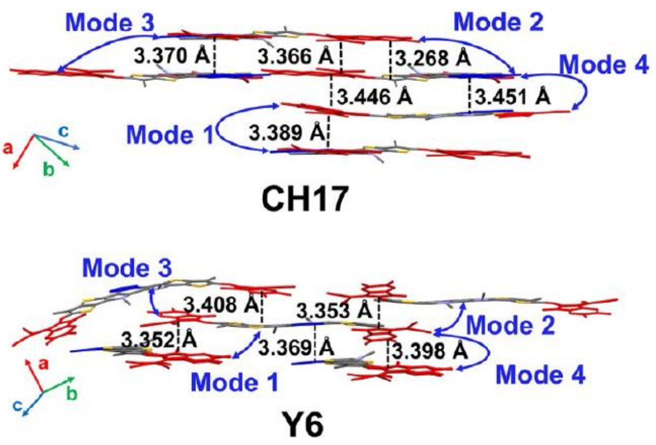

Fig. S20. Intermolecular packing modes of CH17 and Y6 [2].

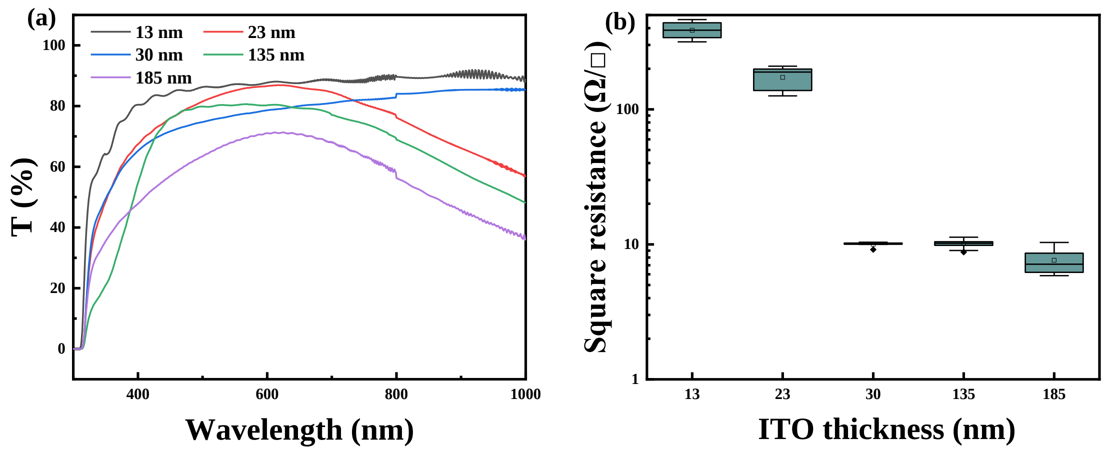

Fig. S21. Characterization of ITO(PET) with different thickness. (a) Transmittance and (b) square resistance.

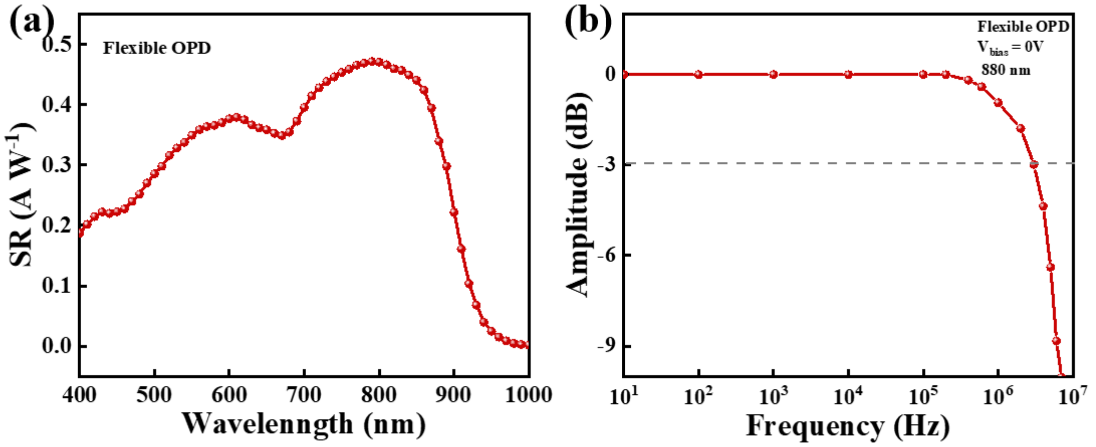

Fig. S22. (a) Responsivity of flexible OPD. (b) -3dB cutoff frequency of flexible OPD (~3 MHz).

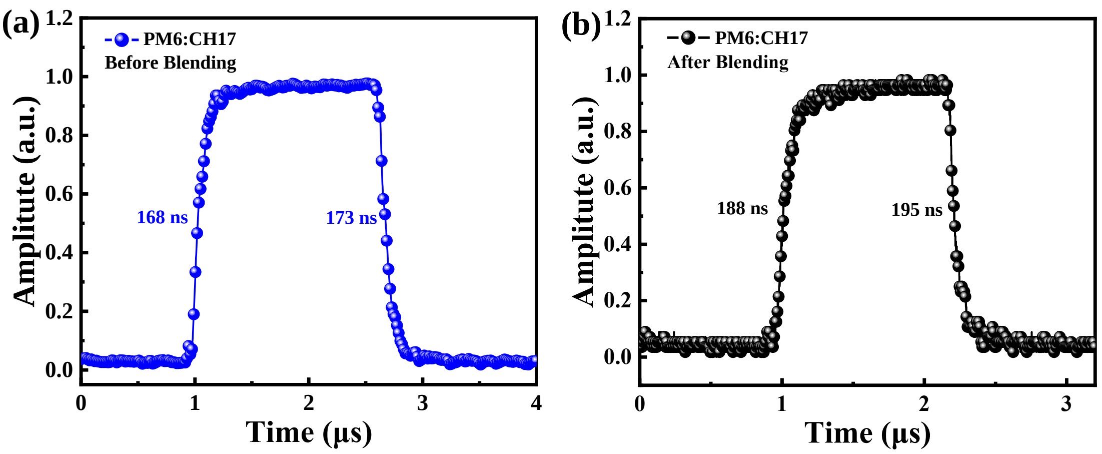

Fig. S23. Response time of PM6:CH17 before (a) and after (b) cycle blending.

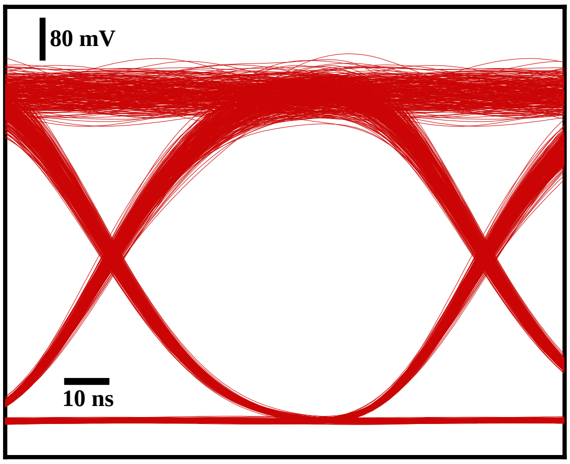

10 Mbps

Fig. S24. Eye diagram at the communication rate of 10 Mbps

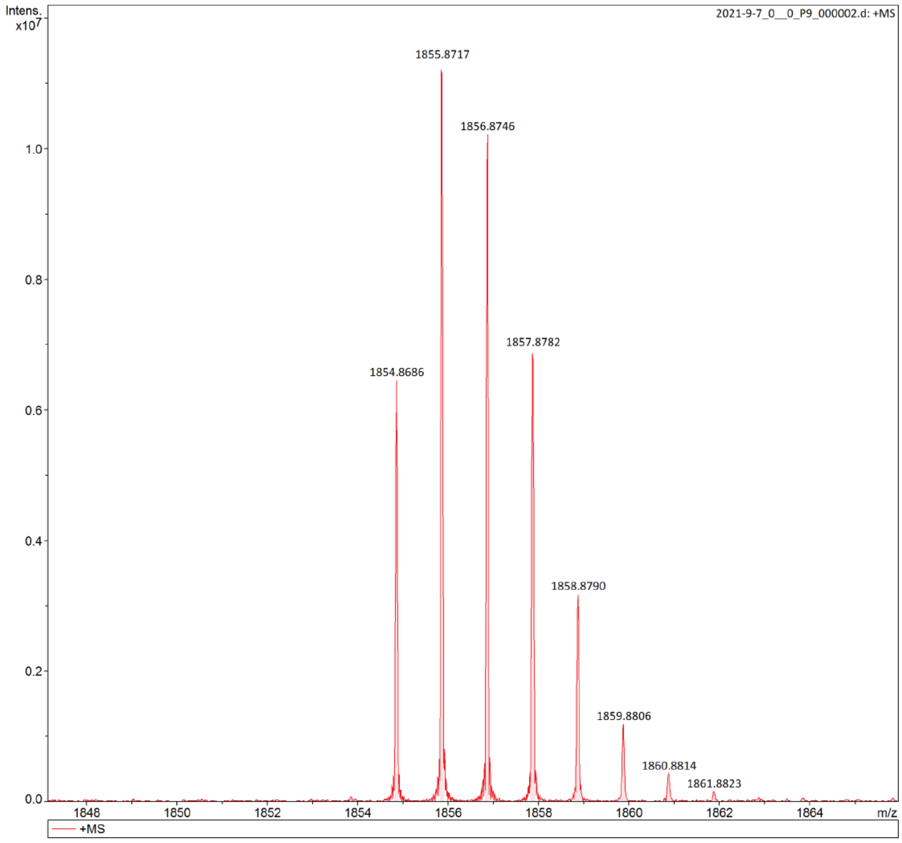

Fig. S25. MS of compound CH17

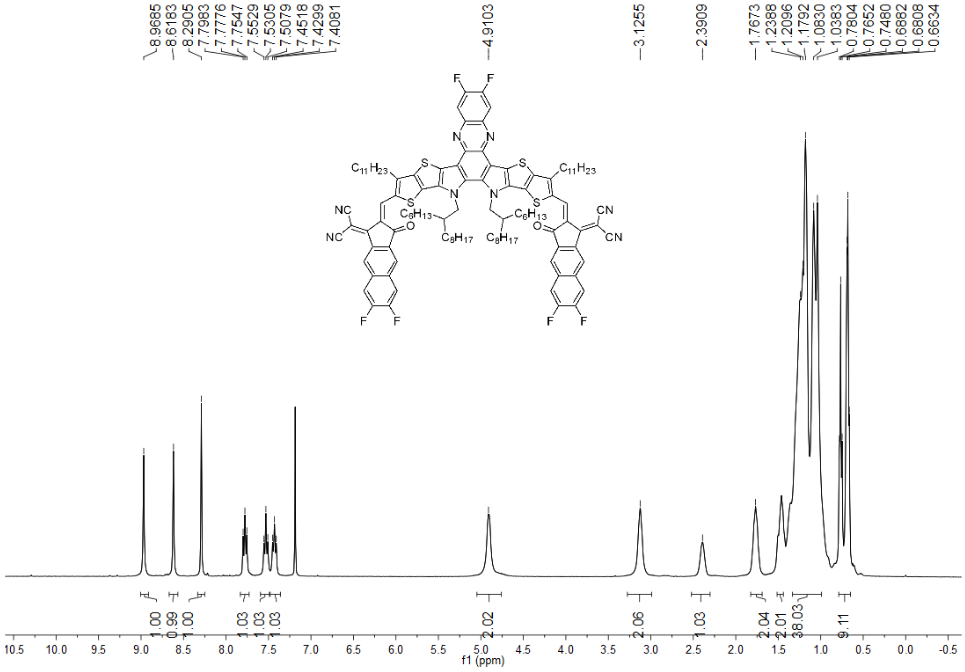

Fig. S26. ^{1}H NMR (400 MHz) spectrum of compound CH17 in $CDCl_3$

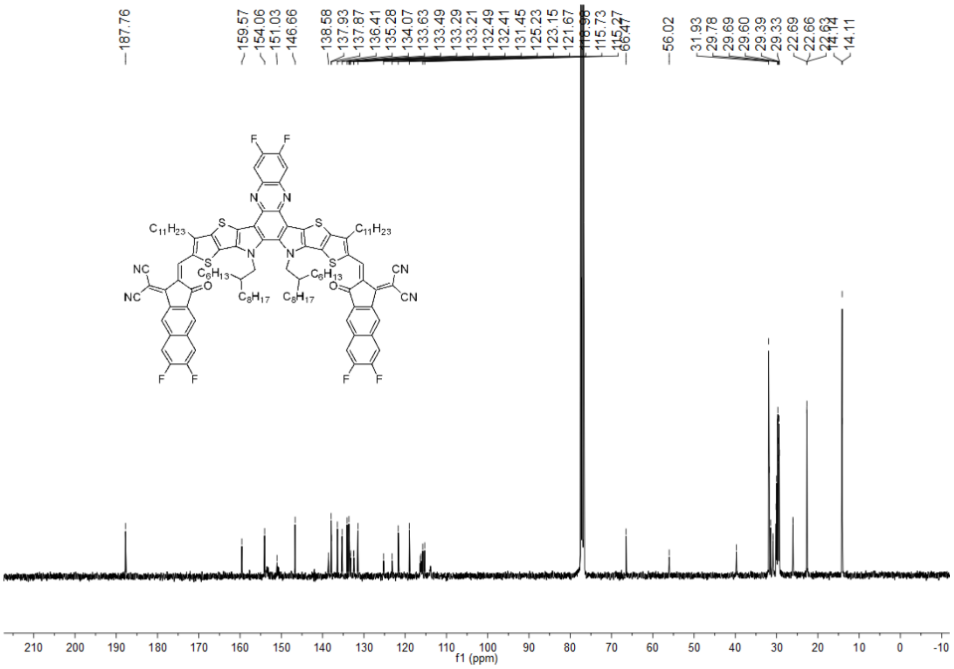

Fig. S27. ^{13}C NMR (100 MHz) spectrum of compound CH17 in $CDCl_3$

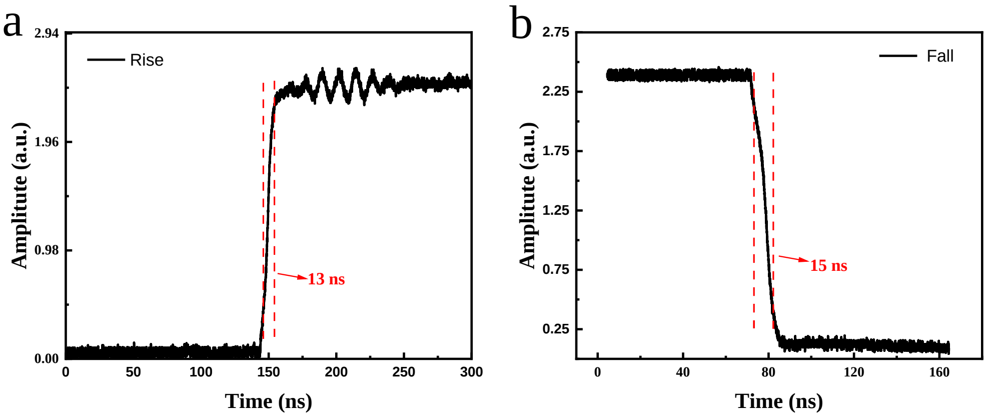

Fig. S28. Response time of 880 nm LED measured by high-speed PIN detector (Hamamatsu S1223)

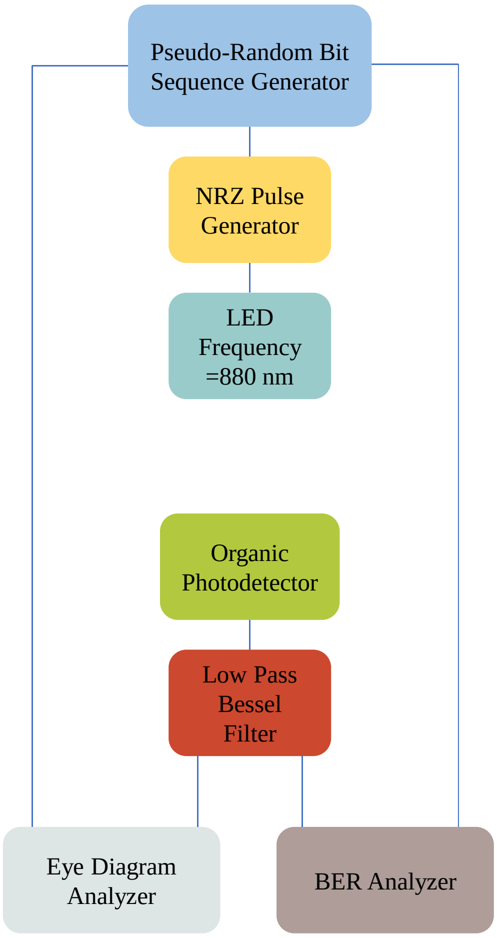

Fig. S29. Simulated flow chart of high-speed optical communication based on OPD at zero bias under 880 nm illumination.

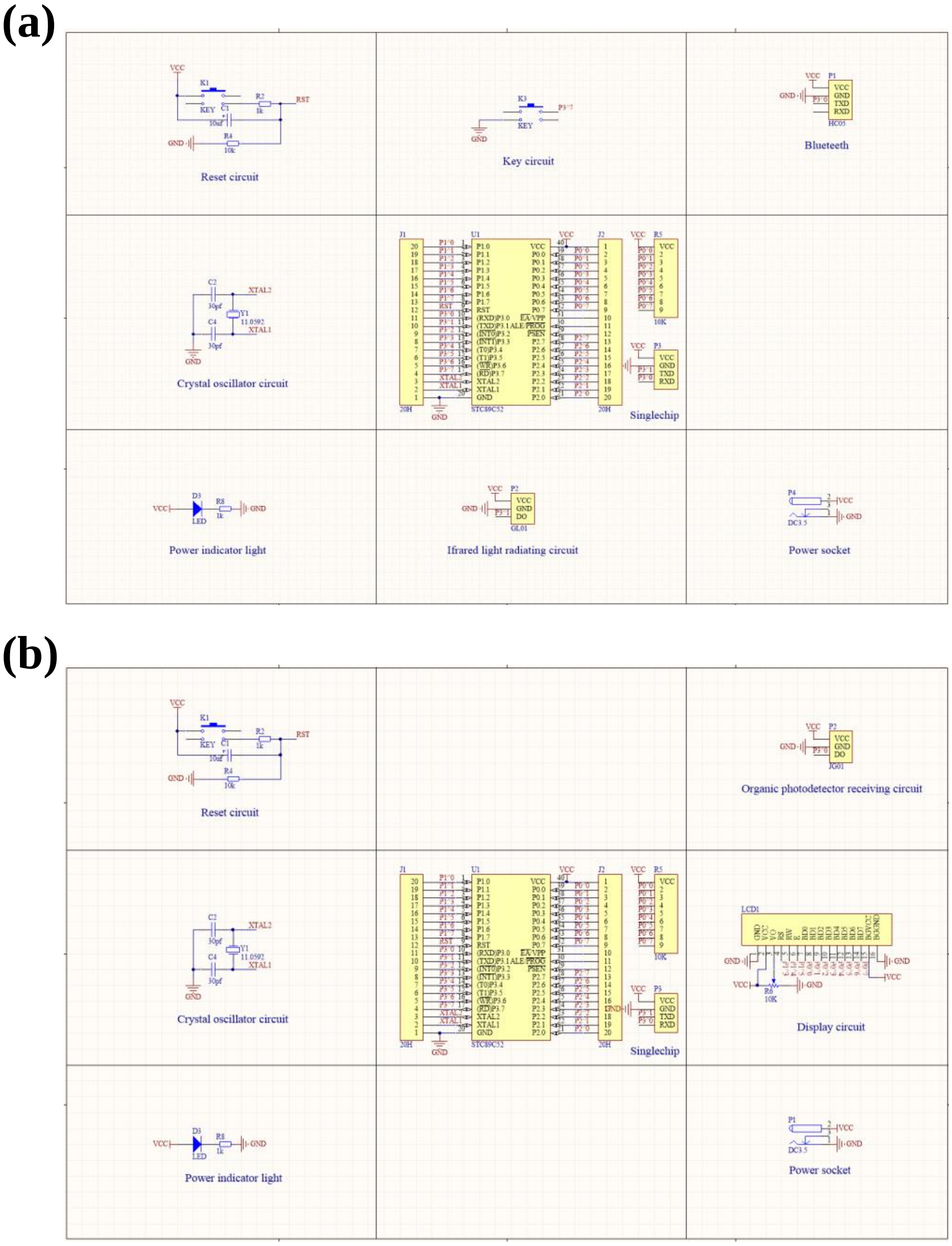

Fig. S30. Circuit diagram of text communication system. (a for Tx-side, b for Rx-side)

Table S1. The specific parameter corresponds to **Fig. 2f.**

	Responsivity (A W^{-1})	Response time (rise time, ns)	Literature/Model
1	0.23 (700 nm)	230	[3]
3	0.35 (700 nm)	900	[4]
4	0.35 (700 nm)	2700	[5]
5	0.45 (950 nm)	1820	[6]
6	0.50 (650 nm)	6200	[7]
2	0.36 (720 nm)	500	S1227-33BQ
7	0.5 (960 nm)	200	S2592-03
8	0.58 (960 nm)	1800	S2387-33R
9	0.6 (960 nm)	1800	S2386-5K
10	0.64 (720 nm)	1500	S16008-33
11	0.64 (960 nm)	1600	S12915-33R
12	0.54 (960 nm)	326	S1133-01
	0.53 (850 nm)	168	This Work

Table S2. Performance of OPD with different thickness

Spin speed (rpm)	Thickness (nm)	Response time (rise time, ns)	Cutoff frequency (MHz)	Responsivity (A/W@850)
500	306	389	1.02	0.37
1000	237	320	1.48	0.51
1500	155	264	2.11	0.57
2000	95	168	3.34	0.53
2500	79	162	3.38	0.39

Table S3. Summary and comparison of flexible photodetectors.

Flexible Substrate	Active layer	Response time	Cutoff frequency	Peak responsivity (A/W)	Ref.	Type
PEN	PCE-10:COTIC-4F	21 μs	-	0.38@940 nm	[8]	OPDs
SU-8/Parylene	PIPCP: $PC_{61}BM$	10 μs	1 kHz	-	[9]	
PES	P3HT:ICBA	35 μs	15 kHz	0.29 @600 nm	[1]	
PI	PEDOT:PSS/C60	5.87 ms	-	0.5 @780 nm	[10]	
PET	PM6:CH17	167 ns	3 MHz	0.48 @830 nm	This Work	
PET	PSC-gated OECT (FET)	67 μs	10 kHz	4.9×10^5 @650 nm (−0.6 V)	[11]	Perovskite PDs
PI	$CsPbI_{3-x}Br_x$	90 μs	-	0.17 @520 nm	[12]	
PET	$CH3NH3PbI_{3-x}Cl_x$	48 ms	-	-	[13]	
PET	CsPbBr3/PbSe	0.5 ms	-	6.26 @400 nm	[14]	
PI	(iBA)2(MA)n−1Pb nI3n+1 film	52 μs	-	0.444 @450 nm	[15]	
PET	CsPbBr3–CsPb2Br5	28 ms	-	0.375 @365nm	[16]	
PC	WS_2	70 μs	5 kHz	0.0027@625 nm	[17]	PDs based on Low-Dimensional Materials
PET	PbS QDs/ ZnO QDs	1.01 s	-	4.54 @375 nm	[18]	
PET	P3HT:CdSe Nanowire	10 ms	-	-	[19]	
PET	PbS/ZnO	9000 ms	-	0.0051 @350nm	[20]	
PET	PbS/PbS	3.63 ms	-	0.10 @1000nm	[21]	

Reference

1. Fuentes-Hernandez C, Chou WF, Khan TM *et al.* Large-area low-noise flexible organic photodiodes for detecting faint visible light. *Science*. 2020; **370**: 698-701.
2. Chen HB, Zou YL, Liang HZ *et al.* Lowing the energy loss of organic solar cells by molecular packing engineering via multiple molecular conjugation extension. *Sci. China Chem.* 2022; **65**: 1362-1373.
3. Tang Z, Ma Z, Sánchez-Díaz A *et al.* Polymer:Fullerene bimolecular crystals for near-infrared spectroscopic photodetectors. *Adv. Mater.* 2017; **29**: 1702184.
4. Li W, Xu Y, Meng X *et al.* Visible to near-infrared photodetection based on ternary organic heterojunctions. *Adv. Funct. Mater.* 2019; **29**: 1808948.
5. Jang W, Rasool S, Kim BG *et al.* Superior noise suppression, response time, and device stability of non-fullerene system over fullerene counterpart in organic photodiode. *Adv. Funct. Mater.* 2020; **30**: 2001402.
6. Huang J, Lee J, Vollbrecht J *et al.* A high-performance solution-processed organic photodetector for near-infrared sensing. *Adv. Mater.* 2020; **32**: 1906027.
7. Lan Z, Lau YS, Wang Y *et al.* Filter-free band-selective organic photodetectors. *Adv. Opt. Mater.* 2020; **8**: 2001388.
8. Simoes J, Dong T, Yang ZC. Non-fullerene acceptor organic photodetector for skin-conformable photoplethysmography applications. *Adv. Mater. Interfaces*. 2022; **9**: 2101897.
9. Park S, Fukuda K, Wang M *et al.* Ultraflexible near-infrared organic photodetectors for conformal photoplethysmogram sensors. *Adv. Mater.* 2018; **30**: 1802359.
10. Hao JX, Wang L, Ma YF *et al.* All-organic thin film flexible photodetector based on C60 modified PEDOT:PSS. *Semicond. Sci. Technol.* 2020; **35**: 085016.
11. Song JJ, Tang GQ, Cao JP *et al.* Perovskite solar cell-gated organic electrochemical transistors for flexible photodetectors with ultrahigh sensitivity and fast response. *Adv. Mater.*: 2207763.
12. Liu ZJ, Li H, Qin CJ *et al.* Solution-processed inorganic perovskite flexible photodetectors with high performance. *Nanoscale Res. Lett.* 2019; **14**: 284
13. Tong GQ, Li H, Li DT *et al.* Dual-phase CsPbBrr3-CsPb2Br5 perovskite thin films via vapor deposition for high-performance rigid and flexible photodetectors. *Small*. 2018; **14**: 1702523.
14. Lai ZX, Meng Y, Zhu Q *et al.* High-performance flexible self-powered photodetectors utilizing spontaneous electron and hole separation in quasi-2d halide perovskites. *Small*. 2021; **17**: 2100442.
15. Wu WQ, Wang XD, Han X *et al.* Flexible photodetector arrays based on patterned ch3nh3pbi3-xclx perovskite film for real-time photosensing and imaging. *Adv. Mater.* 2019; **31**: 1805913.
16. Hu JM, Yang SY, Zhang ZH *et al.* Solution-processed, flexible and broadband photodetector based on CsPbBr3/PbSe quantum dot heterostructures. *J. Mater. Sci. Technol.* 2021; **68**: 216-226.
17. Quereda J, Kuriakose S, Munuera C *et al.* Scalable and low-cost fabrication of flexible WS2 photodetectors on polycarbonate. *npj Flexible Electron.* 2022; **6**: 23.
18. Peng MF, Wang YJ, Shen QQ *et al.* High-performance flexible and broadband photodetectors based on PbS quantum dots/ZnO nanoparticles heterostructure. *Sci. China Mater.* 2019; **62**: 225-235.
19. Wang XF, Song WF, Liu B *et al.* High-performance organic-inorganic hybrid photodetectors based on p3ht:Cdse nanowire heterojunctions on rigid and flexible substrates. *Adv. Funct. Mater.* 2013; **23**: 1202-1209.
20. Zheng Z, Gan L, Zhang JB *et al.* An enhanced uv-vis-nir and flexible photodetector based on electrospun ZnO nanowire array/PbS quantum dots film heterostructure. *Adv. Sci.* 2017; **4**
21. Ren ZW, Sun JK, Li H *et al.* Bilayer pbs quantum dots for high-performance photodetectors. *Adv. Mater.* 2017; **29**: 1702055.
